# DNA Methylation in Neurobiological Genes Associated with Problematic Digital Use in Adolescents and Young Adults: Systematic Review and Exploratory Multilevel Meta-Analysis of Emerging Evidence

**DOI:** 10.3390/epigenomes10030050

**Published:** 2026-08-03

**Authors:** Héctor Cabezas-Klinger, Fabián Felipe Fernández-Daza, Yecid Mina-Paz

**Affiliations:** 1 Grupo de Investigación GIMIA, Facultad de Ciencias Básicas, Universidad Santiago de Cali, Cali 76001, Colombia; hector.cabezas02@usc.edu.co; 2 Facultad de Educación a Distancia y Virtual, Institución Universitaria Antonio José Camacho, Cali 76001, Colombia

**Keywords:** DNA methylation, neurobiological genes, problematic internet use, smartphone, adolescents, young adults, multilevel meta-analysis, emerging evidence

## Abstract

Background: Problematic digital use in adolescents and young adults has emerged as a relevant exposure for mental health and neurobiological vulnerability. However, its relationship with peripheral DNA methylation differences in neurobiologically relevant genes remains poorly synthesized. Objective: This systematic review and exploratory multilevel meta-analysis aimed to synthesize available scientific evidence on associations between phenotypes of problematic internet or smartphone use and DNA methylation differences in neurobiologically relevant genes, and to quantitatively explore the average magnitude of these associations from available data. Methods: Primary human studies were identified through a search strategy with no initial publication-year restriction; the eligible studies retrieved and included in the review were published between 2018 and 2024. The review followed PRISMA criteria and a broad, predefined eligibility strategy to capture both direct and indirect evidence. Seven studies were included in the systematic review; four provided quantifiable data for statistical synthesis, from which 41 effect sizes were harmonized. Analyses were performed in R using multilevel meta-analysis and meta-regression models with REML estimation, supplemented by sensitivity analyses with robust variance estimation. Results: The main meta-analysis revealed a significant positive association between problematic digital exposure and DNA methylation differences (Fisher’s z = 0.2957; r = 0.287; 95% CI [0.1146, 0.4769]; *p* = 0.0020), with high heterogeneity (total I^2^ = 93.15%). Meta-regression indicated that the type of effect and the biological direction of the finding explained a substantial proportion of the observed heterogeneity (total R^2^ ≈ 97.4%). Significance: These findings should be interpreted as exploratory evidence of a biologically plausible association between problematic digital exposure and peripheral DNA methylation differences in neurobiologically relevant genes, rather than as causal or clinically validated biomarker evidence. The available evidence remains methodologically heterogeneous, preliminary, and dependent on a small number of independent studies, underscoring the need for larger, longitudinal, and more methodologically rigorous research in this field.

## 1. Introduction

The rapid expansion of digital technologies has profoundly transformed social, cognitive, and emotional environments, especially during adolescence and early adulthood, stages characterized by high neurobiological plasticity [1,2,3,4]. Heavy or problematic use of the internet, social media, and mobile devices has been identified as an emerging psychosocial stressor associated with anxiety, depression, impulsivity, sleep disturbances, and difficulties in emotional regulation [5,6,7,8,9,10]. These effects are explained, at least in part, by the chronic activation of neurobiological systems involved in the stress and reward response, such as the hypothalamic-pituitary-adrenal (HPA) axis, the dopaminergic system, and socio-emotional circuits, which are particularly sensitive to environmental influences during critical periods of development [1,2,11,12,13]. However, the molecular mechanisms that mediate the relationship between problematic digital use and vulnerability to mental disorders are not yet fully understood.

Epigenetics, and in particular DNA methylation (DNAm), is one of the main biological mechanisms by which environmental exposures can modulate gene expression without altering the DNA sequence [14,15,16,17]. DNAm plays a central role in the regulation of genes involved in stress response, neurotransmission, and emotional regulation, including genes for the HPA axis (NR3C1, FKBP5), the dopaminergic system (SLC6A3, also known as DAT1), serotonergic (SLC6A4, also known as SERT), and oxytocinergic (OXTR), all of which are linked to vulnerability to psychiatric disorders and behavioral alterations [18,19,20]. Evidence suggests that these epigenetic systems act as molecular interfaces that translate environmental exposures into long-lasting functional changes in gene expression and neuronal function, thereby modulating individual susceptibility to mental disorders [21].

Consistent with this model, recent studies have begun to show direct associations between problematic digital use and DNA methylation differences. Annunzi et al. [22] demonstrated that different levels of internet use are associated with differential methylation patterns in key genes such as OXTR, SLC6A3 (DAT1) and SLC6A4 (SERT), in addition to observing direct correlations between the severity of internet use and DNAm levels, suggesting an epigenetic effect dependent on digital exposure. In addition, Annunzi et al. [23] reported significant associations between methylation of the OXTR gene and the severity of internet addiction. Likewise, Li et al. [24] showed that methylation of the circadian PER3 gene moderates the association between problematic mobile phone use and chronotype, indicating that DNAm may act as a biological modulator of susceptibility to problematic digital use. These findings are consistent with the environmental epigenetic programming model, according to which psychosocial exposures can induce long-lasting epigenetic changes in genes involved in emotional regulation and stress response [25,26,27].

Beyond its role as an adaptive mechanism, evidence indicates that environment-induced epigenetic modifications may reflect persistent and potentially transmissible biological processes. Yehuda et al. [20] demonstrated that FKBP5 gene methylation is associated with the clinical course of post-traumatic stress disorder (PTSD) and can be modified in response to therapeutic interventions, confirming the dynamic nature of epigenetic processes. Subsequently, Yehuda et al. [28] showed that traumatic exposures can be associated with persistent epigenetic patterns with intergenerational implications, suggesting that environmental experiences leave lasting molecular imprints [26,27]. These findings provide a robust conceptual framework for investigating whether problematic digital use, as an emerging form of chronic environmental stress, can induce epigenetic modifications with long-term implications for mental health.

Despite biological plausibility and emerging empirical evidence, there is still no systematic and quantitative synthesis that integrates the available evidence on the association between problematic digital use and methylation differences in humans. The current literature presents considerable heterogeneity in terms of population, type of digital exposure, biological tissue analyzed [29,30,31] and reported statistical estimation. This methodological diversity requires robust meta-analytic approaches capable of integrating multiple dependent estimates within the same study, such as multilevel models or robust estimation of variance (RVE) models, which allow estimating global effects without violating the assumptions of statistical independence [32,33]. Therefore, a systematic review and meta-analysis is necessary to quantify the global magnitude of these associations, assess their consistency across neurobiological systems, and identify relevant moderating factors, thus contributing to understanding the biological mechanisms by which the digital environment influences youth mental health. Consequently, this study sets out to answer the following question: what evidence is there on the associations between problematic digital use phenotypes and peripheral DNA methylation differences in neurobiologically relevant genes in adolescents and young adults, and what is the average magnitude of these associations from the available data?

## 2. Objectives

### 2.1. General Objective

To synthesize the available scientific evidence on the associations between problematic digital use phenotypes and peripheral DNA methylation differences in neurobiologically relevant genes in adolescents and young adults, in the context of an emerging line of research, and to quantitatively explore the average magnitude of such associations and their potential moderators from the available data.

### 2.2. Specific Objectives

1. Identify and characterize primary studies evaluating DNA methylation differences in neurobiologically relevant genes in relation to problematic digital use phenotypes, including problematic internet use, problematic mobile phone use, internet addiction, internet gambling disorder, or other empirically operationalized stressful digital exposures.
2. To classify methylation findings according to the neurobiological system involved, including dopaminergic, serotonergic, oxytocinergic, circadian or other relevant systems, and to describe their relationship with internalizing and externalizing psychopathological symptoms, traits or dimensions when these have been evaluated or reported by the included studies.
3. To estimate the average magnitude of associations between phenotypes of problematic digital use and DNA methylation differences, based on studies that report quantifiable measures susceptible to harmonization in effect sizes.
4. To evaluate the heterogeneity of the synthesized associations and to identify possible sources of variability associated with the type of digital exposure, the genetic system involved, the biological direction of the finding, the type of effect measure, the biological tissue analyzed, and the characteristics of the population studied.
5. To explore, through meta-regression and sensitivity analysis, the possible moderating role of the type of biological sample on the synthesized association estimates.

## 3. Methodology

To achieve this objective, the study employs a retrospective systematic review and a meta-analysis, which allows theoretically integrating the most relevant scientific literature in the field and quantitatively synthesizing results on a specific topic [34,35], while identifying conceptual categories and their possible associations.

The protocol for this review was pre-registered with PROSPERO (registration number CRD420261431295).

### 3.1. Procedure

This systematic review and meta-analysis were developed in accordance with the guidelines of the PRISMA 2020 statement (Preferred Reporting Items for Systematic Reviews and Meta-Analyses) to ensure transparency, reproducibility and methodological rigor in the identification, selection and synthesis of scientific evidence [36]. We systematically searched Scopus, PubMed/MEDLINE, Web of Science, Dimensions, Lens, and ScienceDirect, selected for their broad interdisciplinary coverage and relevance in biomedical sciences, neurosciences, and mental health, as well as for their ability to index the epigenetic literature and emerging studies on digital behavior [37,38,39,40].

The search strategy was structured using Boolean operators OR, to expand sensitivity by incorporating conceptual synonyms, and AND, to restrict the results to the thematic intersection of interest. The terms were organized into three main conceptual blocks: (1) epigenetics and methylation, (2) digital stressors and technological behaviors, and (3) young target population. In the first block, the terms methylation, DNA methylation, RNA methylation and epigen were included to capture studies related to quantifiable epigenetic modifications. The second block integrated terms widely used in the literature on digital exposure and mental health, such as problematic internet use, internet addiction, problematic smartphone use, social media addiction, internet gaming disorder, cyberbullying, online victimization, technostress, digital stress, fear of missing out, screen time and related concepts, selected because they represent the most frequent denominations in research on problematic digital use and its psychological and neurobiological correlates [8,10,41]. Finally, the third block included population terms (adolescent, teen, youth, young adult) to limit the search to developmental stages characterized by high neurobiological plasticity and environmental vulnerability [3,4] (Table 1).

In this review, problematic digital use was used as an umbrella term for problematic or addictive patterns involving internet use, smartphone or mobile-phone use, social media, online gaming, cybervictimization, and related digital stressors. The term was used to reflect how exposures were operationalized in the primary studies, not to imply that personal-computer-based internet use and smart-device use are equivalent. A separate device-specific analysis was not feasible because the included studies did not consistently report harmonizable methylation effect sizes by device type.

Among the six databases, 2950 records were identified, with no initial time restriction. Records that met the predefined inclusion and exclusion criteria underwent systematic data extraction (Table 2). Age-related inclusion criteria were defined as adolescents (12–17 years, according to the WHO) and young adults (18–25 years), to encompass stages of high neurobiological plasticity and psychosocial vulnerability. We excluded studies conducted exclusively in adults over 25 years of age, unless they reported disaggregated data for the age ranges of interest, as well as reviews, comments, exclusively animal studies, and research without empirical epigenetic measurements.

**Figure 1:**
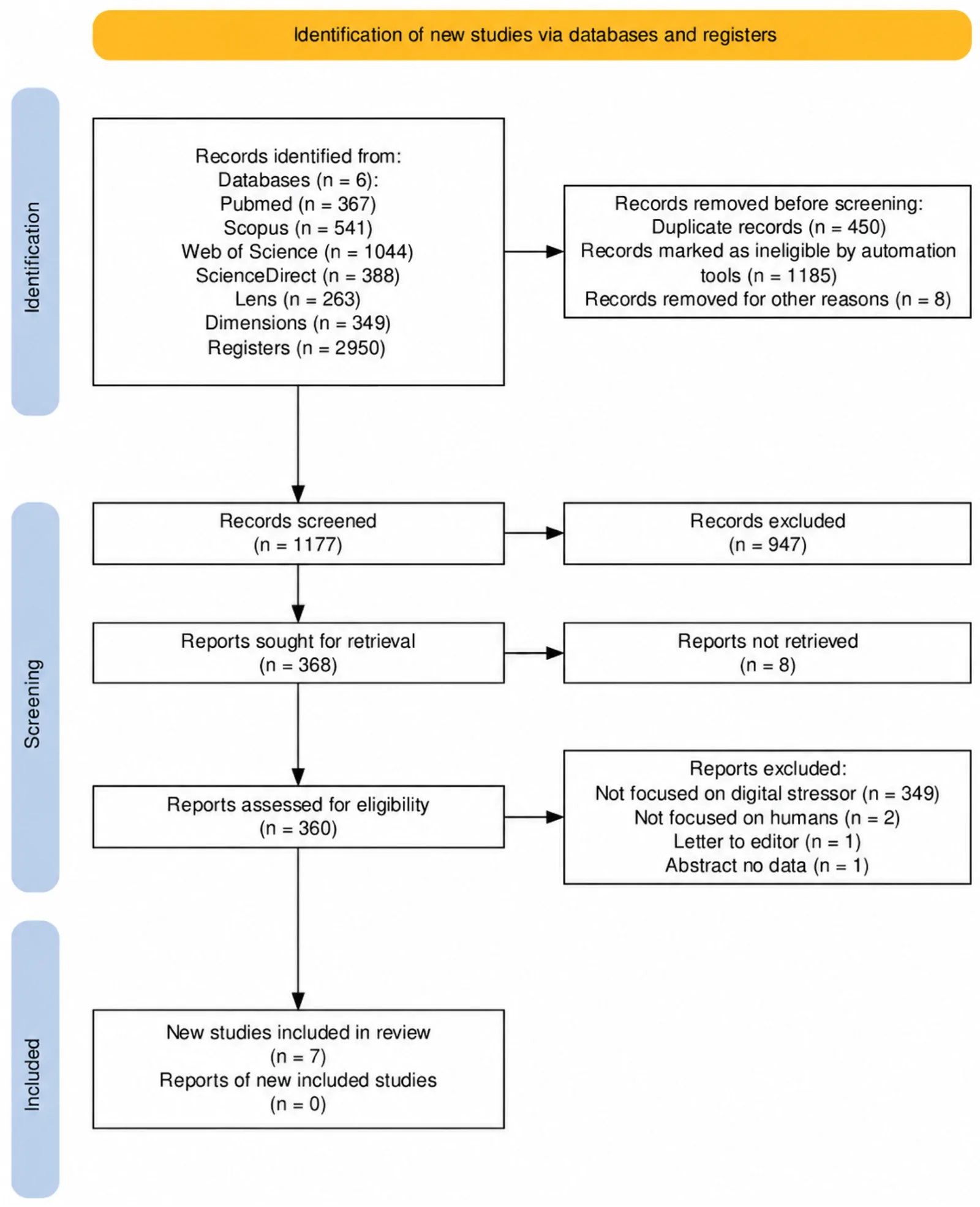
Study selection based on the PRISMA 2020 flow diagram. Figure created by the authors using [42].

A selection process was carried out to eliminate duplicates through automatic review and manual verification, as well as documents irrelevant to the line of research, based on the review of titles, keywords and abstracts, followed by the evaluation of the full text according to the inclusion and exclusion criteria. An audited record was maintained documenting eligibility decisions and reasons for exclusion, ensuring methodological traceability. Figure 1 presents the flowchart detailing the phases of the document selection process, updated in accordance with the PRISMA 2020 standard.

### 3.2. Quantitative Synthesis and Statistical Analysis

The studies included in the quantitative synthesis reported heterogeneous measures of association, including between-group differences, correlations, regression coefficients, and estimates of interaction or moderation. To allow their quantitative integration, the extracted measures were harmonized into a common effect size metric, expressed as a correlation transformed to Fisher’s z when the available data allowed it. For each eligible study, reported measures of association between phenotypes of problematic digital use or empirically operationalized stressful digital exposures and peripheral DNA methylation differences in neurobiologically relevant genes were extracted. When primary studies directly reported effect sizes or statistics sufficient for conversion, they were transformed following standard harmonization procedures. In cases where the authors reported the main effect size but omitted parameters necessary to accurately estimate their variance, standard error, or confidence interval, conservative and prespecified statistical imputations were applied, aimed at avoiding overestimation of the accuracy of the associations. All imputations and assumptions were documented in the Supplementary Material S1, indicating the missing parameter, the assumption applied, and the corresponding justification.

Although seven primary studies met the inclusion criteria for the systematic review, only four provided numerical information that could be harmonized into a common effect-size metric. The meta-analysis was therefore limited to the quantifiable evidence available, while studies with relevant but non-harmonizable findings were retained in the qualitative synthesis and critical appraisal. This approach preserved reproducibility and avoided deriving pooled estimates from inadequately reported data. The concentration of evidence in a small number of countries and research groups was considered a limitation for generalizability.

Because several studies provided multiple effect sizes derived from different genes, CpG sites, outcomes, or laboratory contrasts, statistical independence was not assumed among all estimates. Consequently, multilevel meta-analysis models with random effects were used to model the dependence between effects derived from the same source and to estimate separately the variability between studies and the variability between effects within the study. As a primary sensitivity analysis, robust estimation of variance was also applied to evaluate the stability of the results under an alternative structure of dependence between effect sizes. Additionally, a sensitivity analysis restricted to studies published as full articles was performed, excluding effect sizes from structured conference abstracts when these were represented in the final meta-analytic table, in order to evaluate the stability of the combined effect against the inclusion of preliminary evidence. Results derived from imputed estimates were interpreted with caution and were considered within sensitivity analyses when methodologically relevant. Full statistical software outputs for the multilevel models, robust variance estimation, sensitivity analyses, moderator tests, and tissue-type bootstrap contrasts are provided in the Supplementary Materials S2 and S4 [43,44,45,46].

A sensitivity analysis restricted to studies with clear adjustment for major methylation-related confounders was considered but was not performed as a pooled model, because the number of eligible independent studies and comparable effect-size clusters would be too small for a stable estimate. Instead, confounder adjustment was incorporated into the risk-of-bias assessment and into the interpretation of the pooled effect. The meta-analytic estimate should therefore be read as exploratory rather than as a fully confounder-adjusted causal effect.

## 4. Results

### 4.1. General Characteristics of the Included Studies

We included seven studies published between 2018 and 2024. Five corresponded to full articles and two to structured conference abstracts with minimum extractable quantitative information. Six studies were conducted in adolescents or young adults recruited primarily in university or community youth settings, while one corresponded to a population-based longitudinal cohort of young people assessed up to age 18 [22,23,24,47,48,49,50]. To improve transparency regarding study selection and the evidentiary basis of the synthesis, Table 3 presents a condensed characterization of the included studies, including sample characteristics, psychological or behavioral profile, biological tissue, genes investigated, the main findings, and the role in the synthesis.

Observational analytical cross-sectional designs predominated, with a single exception of longitudinal basis and epigenomic approach. Sample sizes ranged from 32 to 2232 participants; however, direct molecular evidence was mainly concentrated in smaller biological subsamples. Most studies came from Italy, with additional input from China and the United Kingdom [22,23,24,47,48,49,50].

This geographic concentration limits generalizability and should be considered when interpreting the pooled findings, particularly for populations and digital-access contexts not represented in the included studies.

The digital exhibition evaluated was heterogeneous, but conceptually convergent. Five studies focused on problematic internet use or internet addiction [22,23,47,48,49], one examined problematic mobile phone use and chronotype [24], and one assessed victimization throughout development, including cybervictimization, as a form of social stress with potential digital relevance [50]. In all cases, DNA methylation differences were analyzed in peripheral human samples: saliva [22,23,49], buccal swab [47,48], and peripheral blood [24,29,30,31,50].

### 4.2. Qualitative Synthesis

Table 4 presents a synthesis of the main dimensions of finding identified in the included studies, together with the associated epigenetic or biomolecular mechanisms and representative examples of the observed results. Overall, the literature was limited, predominantly candidate-genic, and focused on genes linked to dopaminergic, serotonergic, and oxytocinergic systems, circadian rhythmicity, and stress response [22,23,24,47,48,49,50].

From the integrated reading of the studies, four main axes of results emerged. The first was the association between problematic internet use and DNA methylation differences in genes related to neurotransmission and social-emotional regulation, especially OXTR, SLC6A3 (DAT1) and SLC6A4 (SERT), as reported by Annunzi et al. [23] and Annunzi et al. [22,49]. The second corresponded to behavioral vulnerability profiles, in which methylation or covariation between CpG sites of SLC6A3 (DAT1) was related to impulsivity, attachment, and psychosocial maladjustment [47,48]. The third brought together findings on circadian disruption, where PER3 methylation acted as a modulator of the association between problematic mobile phone use and evening chronotype [24]. The fourth axis was exposure to victimization, including cybervictimization, which showed considerably weaker and less consistent epigenetic evidence [50].

The direction of the findings was not uniform. In the Italian studies on internet addiction, methylation differences were observed in OXTR, SLC6A3 (DAT1) and SLC6A4 (SERT) in a relatively convergent manner; however, the changes did not always follow a linear pattern with the severity of the IAT. In fact, the study in greater molecular detail described an especially marked profile in users at mild risk of problematic use, with methylation increases at specific sites of OXTR and SLC6A3 (DAT1), as well as opposite modifications in SLC6A4 (SERT), suggesting that the epigenetic phenotype could reflect early or transitional states of vulnerability rather than a simple dose–response progression [22,23,49,51].

To organize and synthesize the findings, the psychopathological phenotypes linked to the methylation differences addressed in these studies were grouped within the conceptual framework of internalizing and externalizing spectrums, a robust and empirically validated model that retains its relevance in contemporary psychopathology [52,53]. This dichotomous approach, central to dimensional models such as the Hierarchical Taxonomy of Psychopathology (HiTOP), classifies conditions according to the predominant direction of symptomatic expression: inwards (internalizing; e.g., depression, anxiety, suicidal ideation) or outwards (externalizing; e.g., aggression, impulsivity, norm transgression). This classification is particularly relevant for analyzing the impact of problematic digital use, given that different digital dynamics, for example, social comparison versus cyberbullying, can differentially predispose towards one or the other spectrum.

### 4.3. Internalizing Dimensions

The findings with an internalizing profile focused on processes of emotional regulation, interpersonal sensitivity, social withdrawal, chronodisruption and stress response, rather than on formally established psychiatric diagnoses. This classificatory decision is methodologically reasonable because several studies linked problematic internet use with anxiety, depression, stress, mood disturbances or social concern in their theoretical foundation and, in addition, evaluated genes with clear plausibility for emotional regulation and stress response, such as OXTR, SLC6A4 (SERT) and PER3 [22,24,48,50].

Within this dimension, the oxytocinergic system stood out for its relationship with sociability and the regulation of interpersonal interactions. The preliminary study by Annunzi et al. [23] showed a selective increase in methylation in OXTR CpG4 in students with elevated IAT scores, while the subsequent study by Annunzi et al. [22] identified methylation changes at several OXTR sites, particularly in the group with mild or problematic internet use. Since both studies framed OXTR in processes of social interaction and empathy, these findings can be interpreted as compatible with an internalizing vulnerability related to social sensitivity and emotional regulation in the digital context [22,23].

The serotonergic system also provided signals compatible with internalizing processes. Annunzi et al. [22] identified methylation differences in SLC6A4 (SERT), with inverse correlations with respect to psychometric properties of IAT and with a pattern opposite to that observed in OXTR and SLC6A3 (DAT1). Since the article itself links the regulation of SLC6A4 (SERT) to stress and to behavioral differences versus environmental stressors, these results are plausible in terms of emotional regulation and vulnerability to negative affective states, although they should be interpreted with caution due to the modest sample size and the absence of formal clinical diagnosis [22].

An additional internalizing dimension emerged in the study on chronotype and problematic use of the smartphone. Li et al. [24] showed that problematic mobile phone use was associated with a higher likelihood of evening chronotyping and that PER3 methylation negatively moderated this association. Although the main outcome was not an affective disorder, chronodisruption in young adults is a relevant marker for mental health, emotional regulation, and vulnerability to stress, and the article itself places this finding within the framework of the prevention of circadian rhythm disturbances in young people [24].

Finally, the longitudinal study by Marzi et al. [50] acted as a counterweight within the internalizing-stress dimension. Although it included cybervictimization among the adverse experiences analyzed, it found limited evidence for a robust association between victimization and DNA methylation in peripheral blood. This suggests that the epigenetic response to social or digital stress was not homogeneously detectable in that design and that the epigenetic signal probably depended on the tissue analyzed, the evolutionary moment, and the specificity of the exposure phenotype [50].

### 4.4. Externalizing and Behavioral Dimensions

The externalizing dimension was better represented by studies focused on SLC6A3 (DAT1), impulsivity, problem behavior, and compulsive internet use. In De Nardi et al. [48], the theoretical framework and discussion connected vulnerability to internet addiction with impulsivity, dopaminergic dysregulation, reward-seeking, and attachment difficulties. Empirically, the study showed that the vulnerable subgroup, with high scores in IAT and BIS, presented different patterns of covariation between CpG sites of SLC6A3 (DAT1) 5′-UTR versus controls, especially in the CpG3-CpG5 and CpG5-CpG6 relationships, suggesting that the epigenetic architecture of the dopaminergic system could be linked to a behavioral profile of greater impulsivity and digital risk [48].

Cerniglia et al. [47] reinforced this behavioral axis by studying young people with the SLC6A3 (DAT1) 10/10 genotype and relating internet addiction, impulsivity, and behavioral problems. In that study, the most robust correlations were observed between IAT, BIS scores and behavioral profile variables, including norm transgression, social isolation, intrusiveness, aggressive behavior, anxiety-depression and thought disturbances. At the same time, met5 and met7 methylation loci were correlated with mean SLC6A3 methylation score (DAT1), but multivariate analysis did not demonstrate that SLC6A3 (DAT1) or its alleles significantly predicted psychopathological variables. Therefore, the study supports an externalizing-behavioral constellation associated with problematic internet use, although the direct epigenetic connection to these outcomes was preliminary and weak [47].

Together, these two studies suggest that the dopaminergic axis was the one that showed the greatest conceptual proximity to the externalizing phenomena in this review: impulsivity, reward seeking, control difficulties, relational conflict, and disruptive behaviors associated with problematic internet use. However, the evidence did not indicate a uniform or linear relationship between severity of problematic use and methylation, but rather relational and structural profiles of biological vulnerability [13,47,48].

### 4.5. Emerging Patterns and Underlying Mechanisms

The qualitative analysis allowed us to identify several cross-sectional patterns. First, the evidence suggests that problematic internet and smartphone use is not associated with a single direction of epigenetic change, but with differential methylation profiles according to gene, CpG site, and level of clinical severity. This heterogeneity was especially clear in OXTR, SLC6A3 (DAT1) and SLC6A4 (SERT), where groups with mild or intermediate use came to show more marked DNA methylation differences than groups with higher IAT scores, suggesting nonlinear trajectories of biological vulnerability [22,23,49].

Second, several findings suggest that methylation associated with digital risk may act not only as an outcome, but also as a relational or structural marker. This was observed in SLC6A3 (DAT1) CpG covariation patterns, in which the internal methylation architecture differentiated subgroups defined by psychological vulnerability, genotype, and attachment, rather than by a simple linear gradient of exposure [48]. Similarly, the study on PER3 showed that methylation can play a moderating role, modifying the strength of the relationship between digital exposure and chronotype, rather than simply reflecting it passively [24].

Third, the available literature suggests a functional convergence around neurobiological systems linked to rewards, affective regulation, social interaction, and circadian rhythms. The results on SLC6A3 (DAT1) are consistent with dopaminergic mechanisms of impulsivity, reward deficiency and behavioral dysregulation; the findings on OXTR point to possible processes of social bonding and interpersonal sensitivity; the changes observed in SLC6A4 (SERT) align with emotional regulation and stress response processes; and findings on PER3 connect the digital environment with sleep–wake rhythms and behavioral chronodisruption [13,22,24,47,48].

In contrast, the population-based study on early victimization showed that the association between social adversity and methylation was not easily detectable in peripheral blood when assessed with a broad epigenomic approach and rigorous controls. This suggests that the epigenetic signal associated with digital or social phenomena may critically depend on the phenotype analyzed, the biological tissue, the timing of development, and the degree of proximity between exposure and the biomarker evaluated [50].

### 4.6. Critical Evaluation of the Available Evidence

The available evidence was methodologically limited and dominated by cross-sectional studies, with modest sample sizes in biological subsamples and a marked dependence on candidate-genetic approaches. In addition, several papers came from the same research setting, forcing caution to interpret the apparent convergence of findings, especially in relation to OXTR and SLC6A3 (DAT1) [22,23,48,49]. For the critical evaluation, the risk of bias was adjusted to the design of each study: AXIS was used as the main tool in the complete cross-sectional studies, as it was developed specifically for this type of design and allowed the design, measurement, selection, and quality of the report to be assessed [54]; ROBINS-E was applied to the longitudinal cohort of Marzi et al. [50], since its published version is primarily aimed at follow-up observational studies that estimate the effect of an exposure on an outcome [55]; and structured abstracts were evaluated with AACODS, as they are the gray literature with limited methodological information and are oriented to the assessment of authority, accuracy, coverage, objectivity, date, and significance [56]. Study-level judgments, coding decisions, and the overall risk-of-bias appraisal are provided in Supplementary Material S3 [54,55,56].

In global terms, the main strengths were the use of recognized instruments or scales for digital exposure and psychological variables in 100% of the studies, the availability of sufficient methodological information for a structured assessment in 71.4%, and the explicit declaration of ethical aspects in 71.4%; in contrast, clear control or adjustment for confounding factors were only present in 42.9% of the studies. The overall risk of bias was judged to be moderate, mainly due to non-probabilistic or university-restricted sampling in 85.7% of studies, the use of small or analytically reduced biological subsamples in 71.4%, insufficient or incomplete control for confounders in 57.1%, reliance on self-report-based exposure measures in 100%, and the methodological report necessarily incomplete in the structured abstracts in 28.6%.

Because DNA methylation is sensitive to biological and environmental factors such as age, sex, smoking, alcohol consumption, stress exposure, diet, medication use, and cellular composition, incomplete confounder control remains an important limitation. The available number of clearly adjusted studies was insufficient for a separate sensitivity meta-analysis. This issue was therefore handled through risk-of-bias assessment and cautious interpretation of the pooled effect.

Substantive heterogeneity was also observed in the definition of digital exposure and in the role assigned to methylation. In some studies, it was addressed as the main epigenetic outcome [22,23,49,50], while in others it was analyzed as a dimensional correlate, moderator, or pattern of structural covariation [22,24,47,48]. Overall, although the evidence supports an association between problematic digital use phenotypes and DNA methylation differences in neurobiologically plausible genes, the field remains nascent and requires interpreting the findings as a preliminary basis that warrants larger, longitudinal studies with better characterization of digital exposure [22,24,50].

### 4.7. Meta-Analysis

Of the studies included in the quantitative synthesis [22,23,24,48,49], 41 effect sizes could be harmonized in a common metric and were analyzed using a multilevel random-effects meta-analysis model estimated by REML, implemented in R version 4.5.0 [43] with the metafor and club-Sandwich packages. This strategy is appropriate when multiple effects are nested within the same studies and, therefore, their independence cannot be assumed [57,58].

**Figure 2:**
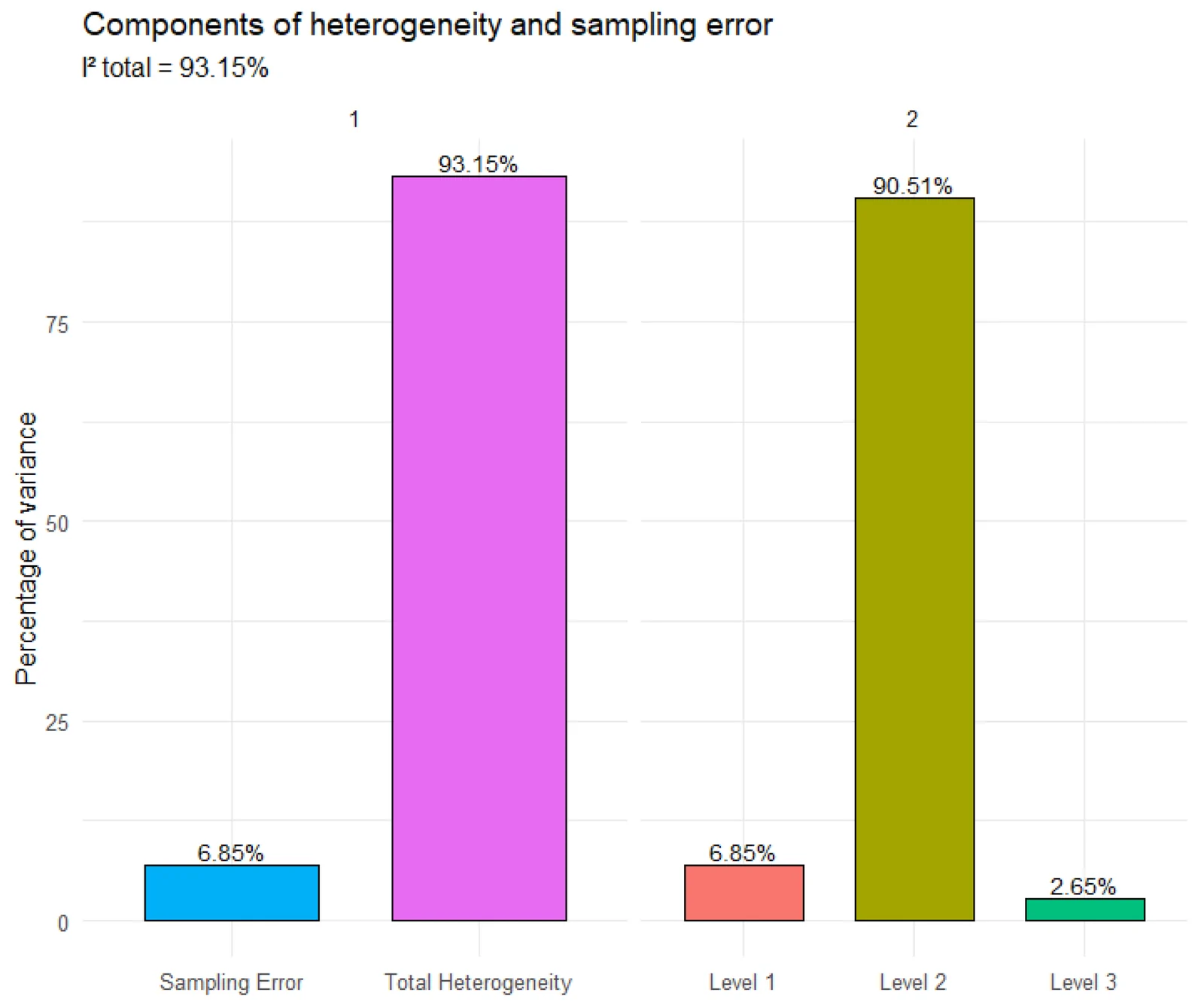
Components of multilevel heterogeneity.

Two components of random variance were specified in the model, one between studies (σ^2^ = 0.0054; τ = 0.0736) and another between effects within the study (σ^2^ = 0.1850; τ = 0.4302), which justifies the designation of the multilevel model and supports its use to manage dependence between effects [57,58]. The combined effect was positive and statistically significant, with an overall estimate in Fisher’s z = 0.2957 (SE = 0.0896; t = 3.2999; df = 40; *p* = 0.0020; 95% CI [0.1146, 0.4769]). Transformed into the correlational metric, this value corresponds to approximately r = 0.287, which, according to contemporary criteria for correlation coefficients, is interpreted as an effect close to the moderate threshold [59,60]. The numerical model outputs are summarized in the text, and the multilevel heterogeneity structure is displayed in Figure 2.

Heterogeneity was significant (Q(40) = 435.9195, *p* < 0.0001). In a multilevel model, this heterogeneity should not be summarized by a single total variance but interpreted from the decomposition of the variance between levels. In this case, the total heterogeneity was very high (total I^2^ = 93.15%), while the remaining 6.85% corresponded to sampling error. Most of the non-sample variance was concentrated in level 2 (I^2^ = 90.51%), i.e., in the variability between effects within the same studies, while the contribution of level 3 was comparatively small (I^2^ = 2.65%), indicating much less heterogeneity between studies. This pattern suggests that much of the observed variability comes from the coexistence of multiple dependent and conceptually distinct effects within the same articles, rather than from marked differences between studies as a whole [57,58].

Consequently, although the combined association differed significantly from zero and points to a positive overall relationship between problematic digital exposure and DNA methylation differences, its magnitude should be interpreted with caution because it summarizes methodologically and biologically diverse evidence; to a large extent, the value obtained is compatible with an effect of moderate magnitude [59,60]. A meta-analysis was also carried out on the same data with robust estimation of variance (RVE). This complementary sensitivity analysis confirmed the positive direction of the finding observed in the multilevel model, although with a slightly more attenuated estimate (r ≈ 0.245) and greater uncertainty.

The combined effect with RVE was Fisher’s z = 0.250 (SE = 0.111; z = 2.251; *p* = 0.024; 95% CI [0.032, 0.467]), with significant heterogeneity (Q(40) = 831.018, *p* < 0.001). The variance between studies was practically zero (σ^2^ = 0.000), while the variance between effects within the study remained high (σ^2^ = 0.217; τ = 0.466), which also suggests that most of the dependence resided in the coexistence of multiple effects within the same articles. Therefore, the RVE estimate is better interpreted as a complementary sensitivity analysis, while the multilevel model retains greater relevance as a primary analysis, by taking advantage of the observed hierarchical structure and offering a more efficient estimate of the combined effect on this specific dataset [58,61].

Influence diagnoses were made at two levels, considering both the study/author clusters and the individual effects [44]. At the cluster level, no studies exceeded the exploratory Cook distance threshold defined as 4/k = 1.0000, so no studies with excessive influence under this criterion were identified. However, the pattern of relative influence was not homogeneous: De Nardi et al. [48] presented the highest Cook distance at the cluster level (Cook’s distance ≈ 0.7843; Absolute maximum DFBETA ≈ 0.7695), followed by Li et al. [24] (Cook’s distance ≈ 0.3018; Absolute maximum DFBETA ≈ 0.5472). In contrast, Annunzi et al. [23] and Annunzi et al. [22] showed low values of influence at the cluster level. At the level of individual effects, most of the effect sizes presented low values of Cook distance; however, localized foci of greater relative influence were observed, mainly in some effects coming from De Nardi et al. [48] and in specific subsets of estimates from Annunzi et al. [22]. Taken together, these results indicate that the combined estimate was not determined by a single individual study or effect, although certain clusters and subsets of effects differentially contributed to the variability of the model. Thus, the influence diagnoses support the interpretation of the meta-analysis as a positive but methodologically heterogeneous synthesis (see Supplementary Material S2).

The joint moderator test was statistically significant (F(7, 34) = 47.284, *p* < 0.001), indicating that these variables explained a substantial proportion of the observed heterogeneity. In quantitative terms, meta-regression explained 100% of the inter-study variance (R^2^ level 3 = 1.00) and approximately 97.4% of the total heterogeneity (total R^2^ = 0.974), with an almost complete reduction in cross-study variance (σ^2^ author: 0.0054 → 0.000) and a marked reduction in between-study variance (σ^2^ author/en.id: 0.1850 → 0.005). However, significant residual heterogeneity persisted (QE(34) = 49.330, *p* = 0.043), indicating that the moderator structure significantly reduced, but not eliminated, the remaining variability of the set [45,62].

The meta-regression coefficients showed that, under the parameterization of the model, the categories EffectTypeCorrelation (b = 0.714, SE = 0.115, *p* < 0.001; 95% CI [0.480, 0.949]) and EffectTypeGroupDiff (b = 0.511, SE = 0.040, *p* < 0.001; 95% CI [0.429, 0.593]) were associated with positive and statistically significant contributions, while the converted EffectTypeBeta did not reach conventional significance (b = −0.078, SE = 0.049, *p* = 0.120). Regarding the biological direction, Hypomethylation (b = −1.009, SE = 0.092, *p* < 0.001), Negative methylation association (b = −1.072, SE = 0.142, *p* < 0.001) and Negative structural coordination (b = −1.319, SE = 0.267, *p* < 0.001) showed significant negative coefficients, while *Positive methylation association* showed a borderline trend (b = −0.241, SE = 0.129, *p* = 0.070). Taken together, these results indicate that the magnitude and sign of the synthesized effects varied systematically by analytic family of effect size and reported epigenetic direction, reinforcing the interpretation that the heterogeneity of the meta-analysis was largely explained by structural differences between outcome types rather than by random fluctuations between studies [45,62].

**Figure 3:**
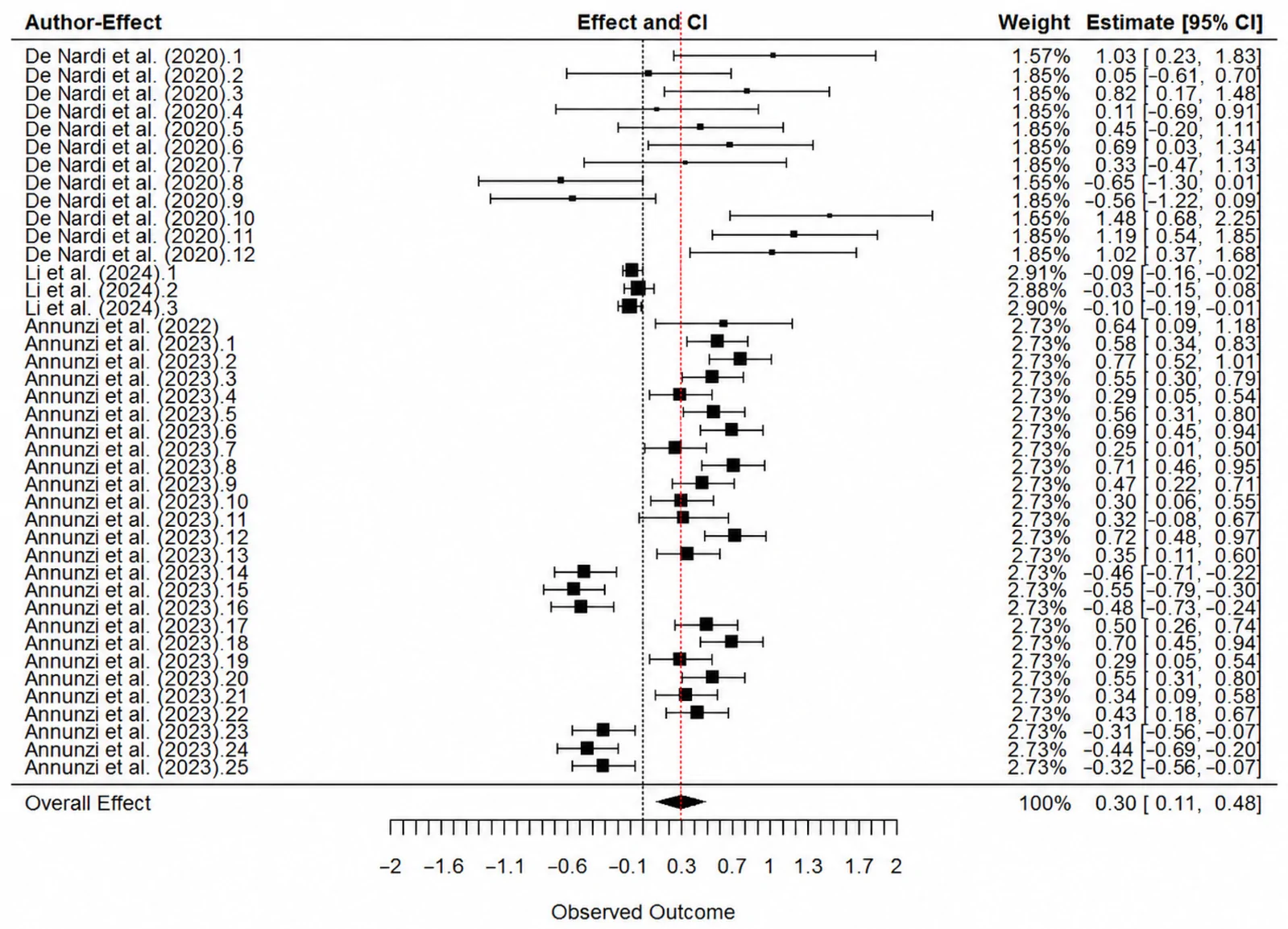
Forest plot of individual effect sizes and pooled effect (Fisher’s z), with 95% confidence intervals. The included studies are: De Nardi et al. [48], Li et al. [24], Annunzi et al. [23] and Annunzi et al. [22]. A total of 41 effects is represented: 12 of De Nardi et al. [48], 3 of Li et al. [24], 1 of Annunzi et al. [23] and 25 of Annunzi et al. [22]. The lower diamond represents the overall pooled effect (z = 0.30; 95% CI [0.11, 0.48]).

Figure 3 presents the forest plot of the 41 effect sizes included in the multilevel meta-analysis model, showing for each effect its point estimate and its 95% confidence interval. Overall, the figure shows a marked dispersion of individual effects, with positive and negative estimates distributed on both sides of the no-effect line, which is consistent with the substantive heterogeneity previously observed. Even so, the combined effect remained in a positive and statistically significant direction (Fisher’s z = 0.30; 95% CI [0.11, 0.48]), since the rhombus corresponding to the global effect does not cross the zero line. This pattern indicates that, when the effects derived from the included studies are considered together, problematic digital exposure is associated with positive average DNA methylation differences, although with marked variability between individual estimates.

### 4.8. Additional Sensitivity Analysis Restricted to Full Articles

In addition to the sensitivity analysis with robust estimation of variance, an additional sensitivity analysis was performed restricting the quantitative synthesis to studies published as full articles. To do this, we excluded the only effect size from a structured conference abstract included in the final meta-analytic table, corresponding to Annunzi et al. [23]. Although Annunzi et al. [49] was considered in the qualitative systematic review, it did not contribute effect sizes to the final quantitative set. Following this exclusion, the analysis included 40 effect sizes.

The multilevel model restricted to complete articles maintained a positive and statistically significant combined estimate (Fisher’s z = 0.2829; SE = 0.1023; t = 2.7655; gl = 39; *p* = 0.0086; 95% CI [0.0760, 0.4899]), roughly equivalent to r = 0.276. Heterogeneity remained significant (Q(39) = 432.297; *p* < 0.0001), with inter-study variance of σ^2^ = 0.0103 and between-study effects variance of σ^2^ = 0.1859. These results indicate that the positive direction and statistical significance of the combined effect did not depend exclusively on the inclusion of preliminary congressional evidence, although substantive heterogeneity remained high. The corresponding technical model output is reported textually rather than as a software screenshot.

Meta-regression restricted to full articles showed a pattern consistent with the main analysis. The co-moderator test was statistically significant (F(7, 33) = 32.5713; *p* < 0.0001), indicating that the type of analytical effect and the biological direction of the finding continued to explain a significant proportion of the variability between effect sizes. Residual heterogeneity remained significant (QE(33) = 48.9651; *p* = 0.0363), although the variance components were significantly reduced compared to the model without moderators.

In terms of approximate reduction in heterogeneity, the total variance went from 0.1962 in the restricted model without moderators to 0.0185 in the restricted model with moderators, equivalent to an approximate reduction of 90.6%. This result supports that the heterogeneity structure identified in the main analysis was not attributable only to the inclusion of the excluded structured abstract but was maintained by restricting the synthesis to complete articles.

### 4.9. Assessment of the Risk of Publication Bias of the Quantitative Synthesis

**Figure 4:**
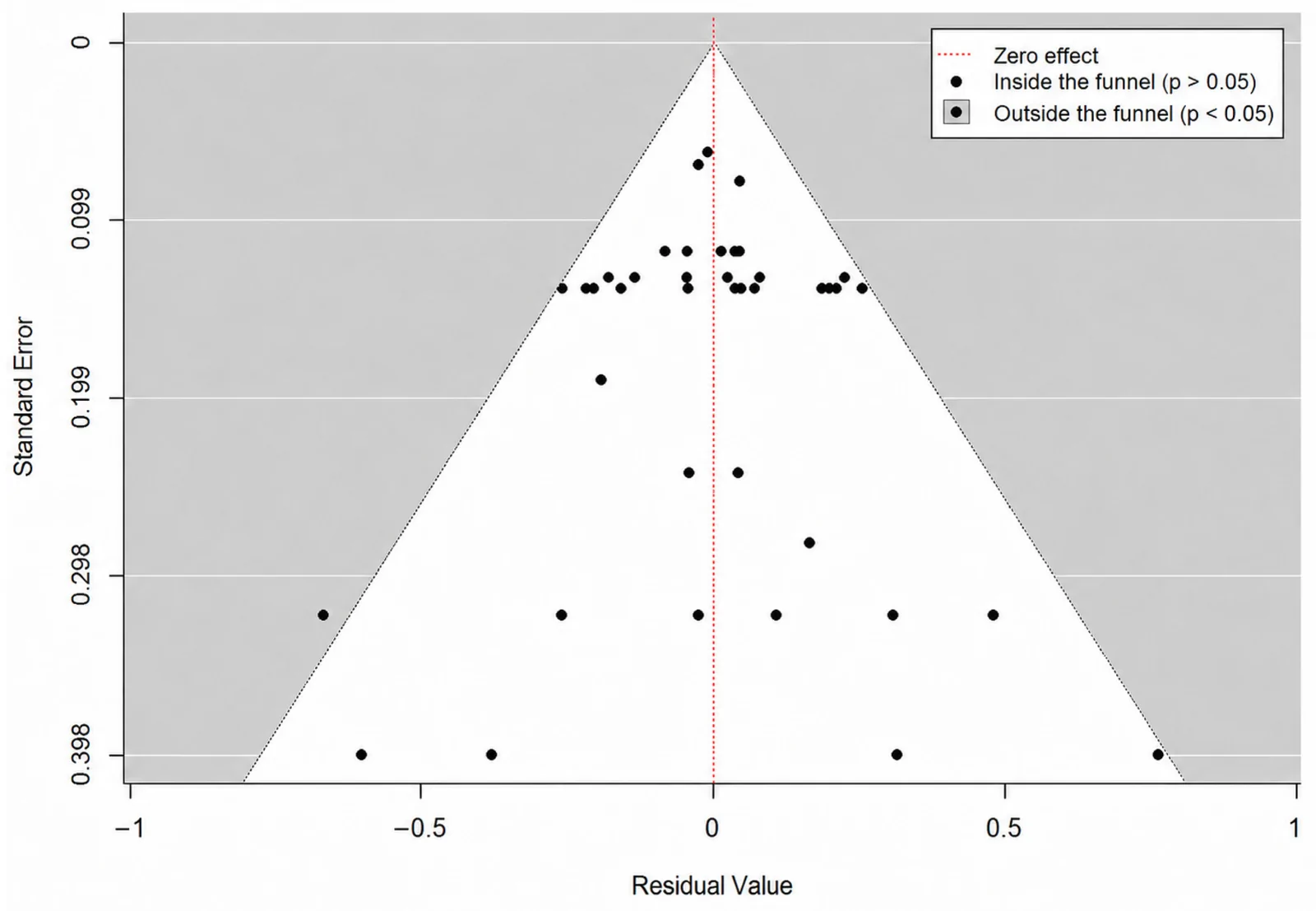
Funnel plot.

Small-study effects and possible publication bias were explored using the funnel plot and Egger regression test [63,64,65,66] (Figure 4). Because the synthesis included few independent studies and several dependent effect sizes, this assessment was interpreted as exploratory. The following hypotheses were evaluated descriptively: H0, no evidence of funnel-plot asymmetry; H1, possible funnel-plot asymmetry, with *p* < 0.05 interpreted as a statistical signal rather than definitive proof of publication bias (Figure 4).

The Egger intercept was not statistically significant (F(1, 2) = 11.3912, *p* = 0.0777), and the funnel plot did not show an unequivocal asymmetry pattern. Given the small number of independent studies and the dependence among several effect sizes, this result should be interpreted cautiously. It indicates that the available data did not confirm a clear small-study-effect pattern, but it does not rule out publication bias.

### 4.10. Hypothesis Testing for Tissue Type

We assessed whether the statistical significance was equivalent between the different types of tissue (levels of the *Tissue* variable). To this end, a method of multiple contrasts was used using bootstrap, implemented with the wildmeta package [46].

The hypotheses were tested as follows:

**H0.**  *The tissue type does not moderate the sizes of the epigenetic effect (β = 0).*

**H1.**  *The tissue type moderates the sizes of the epigenetic effect (β ≠ 0), with rejection of H0 if p < 0.05.*

**Figure 5:**
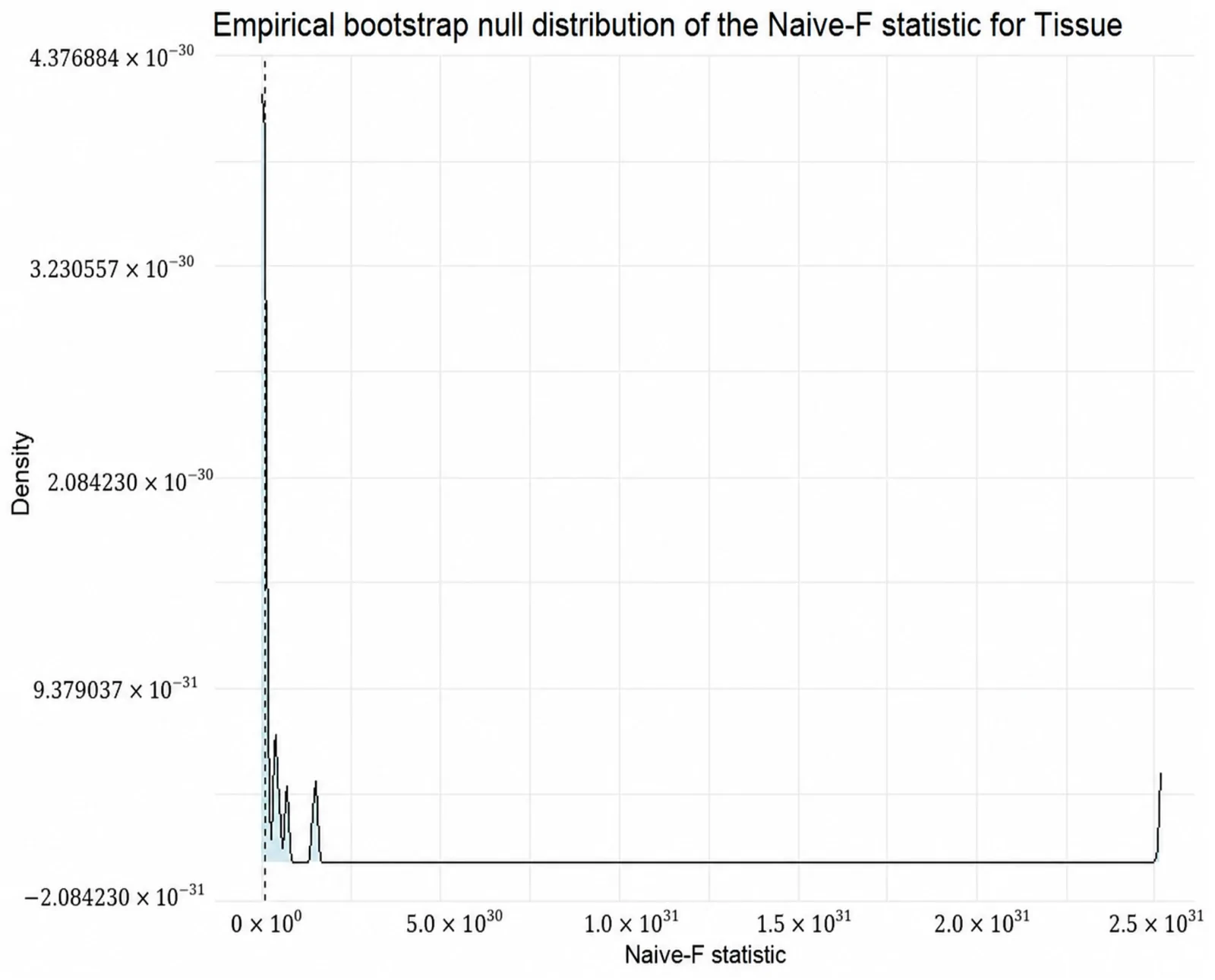
Cluster wild bootstrap Wald test evaluating biological sample type (Tissue) as a moderator of epigenetic effect sizes. The test was performed using 2000 bootstrap replications with CR2 variance correction under the main assumed intra-cluster correlation parameter (ρ = 0.6). The resulting *p*-value (*p* = 0.3665) was above the conventional significance threshold (α = 0.05), indicating that tissue type did not exert a statistically significant moderating effect on the synthesized epigenetic effect sizes.

The result (*p* = 0.3665) indicated that the null hypothesis is not rejected. Therefore, there is no statistically significant evidence that the type of biological sample (Tissue) moderates the sizes of the epigenetic effect in the model evaluated. However, the absence of significant moderation by tissue type should be interpreted with caution and does not constitute evidence of biological equivalence between saliva, buccal swab, and peripheral blood. Figure 5 shows the distribution of the test statistics across all 2000 replicates (see Supplementary Material S4 with rho = 0.6 as a large correlation coefficient and other imputations for this analysis).

## 5. Discussion

This systematic review and exploratory multilevel meta-analysis synthesized the available evidence on the association between problematic digital use phenotypes and peripheral DNA methylation differences in adolescents and young adults. Overall, the quantitative synthesis showed a positive association of small to moderate magnitude (Fisher’s z = 0.2957; r ≈ 0.287), although with substantial heterogeneity between effects and a small number of independent studies. This combination of positive global signal and high heterogeneity supports a biologically plausible but still preliminary association, modulated by important differences in the type of exposure, the analytical function assigned to methylation, and the statistical architecture of the included results (Annunzi et al. [22]; De Nardi et al. [48]; Li et al. [24]).

From a qualitative point of view, the findings converged on four main axes. First, problematic digital exposure was associated with DNA methylation differences in neurotransmission and social-emotional regulation genes, especially OXTR, SLC6A3 (DAT1), and SLC6A4 (SERT) [22,23,49]. Second, several results pointed to a behavioral vulnerability profile linked to the dopaminergic system, in which SLC6A3 (DAT1) methylation or covariation between CpG sites was related to impulsivity, attachment, reward-seeking, and self-regulation difficulties [47,48]. Third, PER3 methylation emerged as a modulator of the association between problematic mobile phone use and evening chronotype, expanding the interpretation of the methylation difference from a mere biological outcome to a moderating function in behavioral and sleep phenotypes [24]. Fourth, the evidence on victimization, including cybervictimization, was less consistent and did not show a robust epigenetic signal in the included population-based longitudinal study [50].

A particularly relevant finding is that methylation did not play a uniform role across studies. In some cases, it was evaluated as the main epigenetic outcome; in others, as a relational marker, association moderator or pattern of structural coordination between CpG sites. This functional diversity has substantial implications: it suggests that epigenetics can not only reflect the biological fingerprint of digital exposure but also modulate individual susceptibility and organize neurobehavioral vulnerability profiles. At the same time, this heterogeneity complicates direct comparability between studies and explains much of the variance observed in the meta-analysis.

The direction of the findings was also not linear. In Italian studies on internet addiction, the group with mild or intermediate use came to show more marked DNA methylation differences than the group with higher IAT scores, especially in OXTR and SLC6A3 (DAT1) [22]. This suggests that DNA methylation differences could reflect early or transitional states of vulnerability, rather than a simple dose–response relationship with the severity of problematic use. Similarly, studies on SLC6A3 (DAT1) showed that digital risk could be related not only to the level of exposure, but also to the internal organization of the epigenetic signal, to CpG coordination patterns linked to impulsivity, genotype, and attachment quality [48].

From a psychobiological perspective, the results are consistent with the involvement of well-established functional systems in the literature on adolescence, stress, and emotional regulation. SLC6A3 (DAT1) aligns with dopaminergic processes of reward, impulsivity, and behavioral control; OXTR, with social bonding and interpersonal sensitivity; SLC6A4 (SERT), with emotional regulation and stress response; and PER3, with sleep–wake rhythms and chronodisruption. Taken together, this convergence suggests that the problematic digital environment may interact with sensitive biological systems in developmental stages characterized by high neurobehavioral plasticity, such as adolescence and young adulthood [24,47,48].

The biological interpretation of peripheral DNA methylation also requires caution. Saliva, buccal cells, and peripheral blood are accessible tissues for human epigenetic research, but they are not direct measures of brain methylation. Cross-tissue evidence suggests that some methylation signals may show partial covariability or psychiatric relevance, whereas many methylation patterns remain tissue-specific and cell-type-dependent [29,30,31]. Accordingly, the present findings are best interpreted as peripheral epigenetic correlates in neurobiologically relevant genes, not as direct evidence of brain methylation status.

However, the evidence base remains incipient. Cross-sectional designs, non-probabilistic university samples, small biological subsamples, peripheral tissues, and candidate-gene approaches predominated. In addition, several studies came from the same research setting, which requires caution when interpreting the apparent convergence of results in OXTR and SLC6A3 (DAT1) [22,23,24,48,49]. Critical reappraisal showed a moderate overall risk of bias, mainly due to restricted sampling, the use of self-reports, and insufficient control for confounders.

The predominance of cross-sectional designs also prevents establishing temporal ordering. The available evidence cannot determine whether problematic digital use preceded methylation differences, whether pre-existing epigenetic or neurobehavioral vulnerability increased susceptibility to problematic use, or whether both reflected shared environmental and psychological factors. Accordingly, the findings are framed as preliminary associations and peripheral epigenetic correlates, not as evidence that digital use causes DNA methylation changes.

The synthesis also does not assume that all devices or platforms operate through identical mechanisms. Internet use, smartphone or mobile-phone use, social media exposure, gaming, and cybervictimization may differ in access, portability, reward patterns, social interaction, and sleep disruption. A separate comparison between personal-computer-based use and smartphone or smart-device use was not feasible because the included studies did not provide enough device-specific methylation effect sizes. Future studies should distinguish platform, device, duration, context, and compulsiveness of use.

An additional limitation of quantitative synthesis was that some primary studies did not report all the parameters needed to directly calculate variance, standard error, or confidence intervals for certain effect sizes. Although conservative and documented statistical imputations were applied to preserve analytical transparency and avoid overestimation of accuracy, these decisions introduce an additional degree of uncertainty. Therefore, the results of the meta-analysis should be interpreted as an exploratory estimate based on the best available quantitative information, rather than as a confirmatory synthesis of fully reported homogeneous effects.

Quantitatively, heterogeneity was very high and was almost entirely concentrated in the variability between effects within the study. This finding is consistent with the coexistence of multiple conceptually distinct effect sizes drawn from a small number of articles and supports the decision to use multilevel models and sensitivity analyses with robust estimation of variance. Additionally, sensitivity analysis restricted to full articles maintained the positive direction and statistical significance of the combined effect, reducing the possibility that the overall signal relies exclusively on preliminary congressional evidence. Meta-regression showed that the type of effect and the biological direction of the finding explained much of the heterogeneity, indicating that much of the observed dispersion was due to structural differences between the synthesized results and not exclusively to random error.

Although no statistically conclusive evidence of publication bias was detected, this absence of significance should be interpreted with caution, given the small number of studies and the dependence between effects. Consequently, the results of the Egger test and the funnel plot are not considered here as conclusive evidence of the absence of bias, but rather a lack of robust evidence to confirm it.

In terms of correspondence with the specific objectives, the review allowed us to identify and characterize the available primary studies; organize the findings according to dopaminergic, serotonergic, oxytocinergic and circadian neurobiological systems; estimate an average magnitude of association using multilevel models; assess heterogeneity and its sources using meta-regression; and explore the possible moderating role of the type of biological sample. This correspondence should not be interpreted as a definitive confirmation of the proposed mechanisms, but as an analytical fulfillment of the objectives set under an exploratory framework and with the limitations of emerging evidence.

Overall, this systematic review and exploratory multilevel meta-analysis support the existence of a preliminary association between problematic digital exposure and peripheral DNA methylation differences in neurobiologically plausible genes, but they do not establish robust causal trajectories or stable biomarkers of risk. The biological signal is suggestive, coherent, and methodologically defensible within the limits of emerging evidence; however, it remains preliminary and should not be interpreted as proof that problematic digital use induces methylation changes. Therefore, the findings should be understood as an emerging evidence base that justifies broader, longitudinal investigations, with better characterization of digital exposure, pre-specified analytical strategies, and external validation.

## 6. Conclusions

Currently available evidence suggests that problematic digital use phenotypes in adolescents and young adults are associated with peripheral DNA methylation differences in genes involved in neurotransmission, emotional regulation, circadian rhythms, and stress response. The multilevel meta-analysis showed a positive and significant combined effect, of small to moderate magnitude, while the meta-regression showed that much of the heterogeneity was explained by the type of analytical effect and the biological direction of the finding. In its central aspect, this suggests that the relationship between problematic digital exposure and DNA methylation is not uniform, but depends on the gene studied, the role assigned to methylation, and the psychological or behavioral context in which it is evaluated.

Despite this, the field remains methodologically immature. The predominance of cross-sectional studies, small biological samples, peripheral tissues, reliance on self-reports, limited control of confounders, and concentration of evidence in a few countries and research groups limit generalization and causal inference. Consequently, the results should be interpreted as preliminary and biologically plausible evidence based on peripheral methylation markers, rather than as definitive causal, brain-equivalent, or clinically validated biomarker evidence. These findings justify longitudinal, multicenter, device-specific, and analytically more robust studies.

## 7. Recommendations

### 7.1. For Research

Longitudinal studies with repeated measurements of digital exposure and epigenetic biomarkers are required to better discriminate between prior vulnerability, selection effect, and digital exposure effect. It is also a priority to broaden the focus beyond candidate-genetic approaches, incorporate independent validation samples and more accurately characterize digital exposure, differentiating intensity, compulsiveness, type of platform, time pattern and relational context of use. Similarly, it is advisable to integrate variables of emotional regulation, sleep quality, attachment, impulsivity and perceived stress in prespecified multivariate models.

### 7.2. For Methodology and Analysis

Future quantitative syntheses should be planned from the outset to manage dependence between multiple effects per study, ideally using multilevel models or robust sensitivity strategies. It is also advisable to standardize the way epigenetic results are reported, including n by subgroup, standard errors, confidence intervals, and conversion parameters, to facilitate comparability between studies and the reproducibility of meta-analyses.

### 7.3. For Clinical and Preventive Practice

Although the evidence does not yet justify the clinical use of epigenetic markers, it does support the need to address problematic digital use as a phenomenon relevant to youth mental health, especially when it coexists with impulsivity, sleep disruption, emotional regulation difficulties, or experiences of victimization. In this context, preventive interventions should strengthen critical digital literacy, emotional regulation, sleep hygiene, and family and school support, rather than focusing exclusively on restricting technological access.

## Figures and Tables

**Table 1:** Search expressions used in the databases queried.

Database	Search Strategy
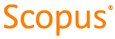	(TITLE-ABS-KEY (methylation* OR “DNA methylation*” OR “RNA methylation” OR epigen*) AND TITLE-ABS-KEY (“problematic internet use” OR “internet addiction” OR “internet use disorder” OR “problematic smartphone use” OR “smartphone addiction” OR “problematic social media use” OR “social media addiction” OR “internet gaming disorder” OR “cyberbullying” OR “online victimization” OR “online harassment” OR “digital peer victimization” OR “technostress” OR “digital stress” OR “fear of missing out” OR “social media” OR “social comparison” OR “problematic social network* use” OR “problematic use of social networking sites” OR “addictive social media use” OR “social media-related stress” OR “social media fatigue” OR “online social overload” OR “cell phone” OR “mobile phone” OR “smartphone” OR “technology addiction” OR “mobile application*” OR “time perception” OR “selective dissemination of information” OR “social media exposure” OR “anxiety” OR “social network*” OR “social media” OR “impulse control disorders” OR “emotional regulation” OR “media exposure” OR “screen time” OR cyberbullying) AND TITLE-ABS-KEY (adolescent* OR teen* OR youth* OR “young adult*”))
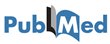	(methylation* OR “DNA methylation*” OR “RNA methylation” OR epigen*) AND (“problematic internet use” OR “internet addiction” OR “internet use disorder” OR “problematic smartphone use” OR “smartphone addiction” OR “problematic social media use” OR “social media addiction” OR “internet gaming disorder” OR “online victimization” OR “online harassment” OR “digital peer victimization” OR “technostress” OR “digital stress” OR “fear of missing out” OR “social media” OR “social comparison” OR “problematic social network* use” OR “problematic use of social networking sites” OR “addictive social media use” OR “social media-related stress” OR “social media fatigue” OR “online social overload” OR “cell phone” OR “mobile phone” OR “smartphone” OR “technology addiction” OR “mobile application*” OR “time perception” OR “selective dissemination of information” OR “social media exposure” OR “anxiety” OR “social network*” OR “social media” OR “impulse control disorders” OR “emotional regulation” OR “media exposure” OR “screen time” OR cyberbullying) AND (adolescent OR teen OR teenager OR youth OR “young adult”)
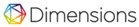	(methylation OR “DNA methylation” OR “RNA methylation” OR epigenetics OR “CpG” OR Histones) AND (“problematic internet use” OR “internet addiction” OR “internet use disorder” OR “problematic smartphone use” OR “smartphone addiction” OR “problematic social media use” OR “social media addiction” OR “internet gaming disorder” OR “cyberbullying” OR “online victimization” OR “online harassment” OR “digital peer victimization” OR “technostress” OR “digital stress” OR “fear of missing out” OR “social media” OR “social comparison” OR “problematic social network use” OR “problematic use of social networking sites” OR “addictive social media use” OR “social media-related stress” OR “social media fatigue” OR “online social overload” OR “cell phone” OR “mobile phone” OR “smartphone” OR “technology addiction” OR “mobile application” OR “time perception” OR “selective dissemination of information” OR “social media exposure” OR “anxiety” OR “social network” OR “social media” OR “impulse control disorders” OR “emotional regulation” OR “media exposure” OR “screen time”) AND (adolescent OR teen OR teenager OR youth OR “young adult”)
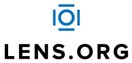	(methylation* OR “DNA methylation*” OR “RNA methylation” OR epigen* OR CpG OR Histones) AND (“problematic internet use” OR “internet addiction” OR “internet use disorder” OR “problematic smartphone use” OR “smartphone addiction” OR “problematic social media use” OR “social media addiction” OR “internet gaming disorder” OR cyberbullying OR “online victimization” OR “online harassment” OR “digital peer victimization” OR technostress OR “digital stress” OR “fear of missing out” OR “social media” OR “social comparison” OR “problematic social network* use” OR “problematic use of social networking sites” OR “addictive social media use” OR “social media-related stress” OR “social media fatigue” OR “online social overload” OR “cell phone” OR “mobile phone” OR smartphone OR “technology addiction” OR “mobile application*” OR “time perception” OR “selective dissemination of information” OR “social media exposure” OR anxiety OR “social network*” OR “social media” OR “impulse control disorders” OR “screen time” OR “emotional regulation” OR “media exposure”) AND (adolescent* OR teen* OR youth* OR “young adult*”)
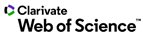	(methylation* OR “DNA methylation*” OR “RNA methylation” OR epigen*) AND (“problematic internet use” OR “internet addiction” OR “internet use disorder” OR “problematic smartphone use” OR “smartphone addiction” OR “problematic social media use” OR “social media addiction” OR “internet gaming disorder” OR “online victimization” OR “online harassment” OR “digital peer victimization” OR “technostress” OR “digital stress” OR “fear of missing out” OR “social media” OR “social comparison” OR “problematic social network* use” OR “problematic use of social networking sites” OR “addictive social media use” OR “social media-related stress” OR “social media fatigue” OR “online social overload” OR “cell phone” OR “mobile phone” OR “smartphone” OR “technology addiction” OR “mobile application*” OR “time perception” OR “selective dissemination of information” OR “social media exposure” OR “anxiety” OR “social network*” OR “social media” OR “impulse control disorders” OR “emotional regulation” OR “media exposure” OR “screen time” OR cyberbullying) AND (adolescent OR teen OR teenager OR youth OR “young adult”)
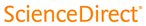	(Find articles with these terms):(epigenetics OR epigenomics OR methylation OR “DNA methylation” OR “RNA methylation”) AND (Title, abstract or author-specified keywords):(“Problematic Internet Use” OR “Internet Addiction” OR “Internet Use Disorder” OR “Problematic Smartphone Use” OR “Smartphone Addiction” OR “Problematic Social Media Use” OR “Social Media Addiction” OR “Internet Gaming Disorder” OR “Cyberbullying”) AND (adolescent OR teen OR teenager OR youth OR “young adult”)

Note. The asterisk (*) is a truncation wildcard used in search queries to include multiple word endings. For example, searching for ‘disorder’ retrieves records containing ‘disorder’ and ‘disorders’, and adolescent* gets ‘adolescent’, ‘adolescents’, and ‘adolescence’.

**Table 2:** Inclusion and exclusion criteria for the review.

Inclusion Criteria	Exclusion Criteria
Original article, case study, brief communications and structured abstracts (congress abstracts) with minimum extractable quantitative information.	Non-original article, no case study or document without original data.
Applicable to teens and young adults.	Applied to another age group.
Focused on digital stressors (problematic use of the internet, mobile phone, or equivalent digital interaction).	Not focused on problematic use of the internet, mobile phone, or equivalent digital interaction.
Human studies.	Studies in non-humans.
Reporting of differences in DNA methylation differences associated with problematic digital use phenotypes or empirically operationalized stressful digital exposures.	No DNA methylation differences associated with phenotypes of problematic digital use or empirically operationalized stressful digital exposures were reported.
Written in English or another language with English translation available.	No English translation available.
Full access to the publication.	No access to the full publication.

**Table 3:** Condensed characteristics of the studies included in the systematic review.

Study	Design/ Publication Type	Sample and Recruitment	Psychological/ Behavioral Profile Assessed	Tissue/ Biological Sample	Genes or Methylation Targets	Main Findings	Role in Synthesis
De Nardi et al. [48]	Cross-sectional candidate-gene study; full article.	Community youth sample from Italy; n = 79 (20 males, 59 females), age range 18–34 years, mean age approximately 23 years; three a posteriori analytic subgroups were derived: one vulnerable high-IAT/high-BIS subgroup (n = 9) and two low-impulsivity control subgroups stratified by DAT1 genotype and secure attachment pattern (n = 12 each).	Internet Addiction Test, Barratt Impulsiveness Scale, and attachment-related relational quality.	Buccal swab DNA.	SLC6A3/DAT1, including 5′-UTR methylation and 3′-UTR VNTR genotype.	DAT1 methylation covariation patterns differed across vulnerability/control profiles, suggesting an interaction among dopaminergic epigenetic architecture, impulsivity, attachment, and problematic internet-use tendency.	Included in qualitative synthesis and quantitative meta-analysis.
Li et al. [24]	Cross-sectional observational study; full article.	Young adults recruited from two Chinese universities; 1179 selected, 1135 valid questionnaires, and 744 blood samples for PER3 methylation assessment.	Problematic mobile phone use and chronotype; sex-stratified analyses.	Peripheral blood.	PER3.	Problematic mobile phone use was associated with evening chronotype, and PER3 methylation showed a negative moderating role in this association, with sex-related differences.	Included in qualitative synthesis and quantitative meta-analysis.
Annunzi et al. [49]	Structured conference abstract with extractable quantitative information.	Young adult university sample; detailed age and sex distribution were limited by the abstract format.	Internet Addiction Test/internet-addiction severity.	Peripheral DNA source reported in abstract-level material; limited methodological detail.	SLC6A3/DAT1.	Preliminary evidence of DAT1 methylation differences in relation to high internet-addiction scores.	Included in qualitative synthesis; quantitative contribution only if extractable effect-size information is retained in the final dataset.
Annunzi et al. [22]	Cross-sectional candidate-gene study; full article.	University students from Italy; n = 84, including 20 males and 64 females; mean age 20.1 ± 2.09 years.	Internet Addiction Test, with groups defined as controlled use, mild-risk use, and moderate/problematic use.	Salivary DNA.	OXTR, SLC6A3/DAT1, and SLC6A4/SERT.	Mild-risk internet users showed significant methylation differences in OXTR, DAT1, and SERT, with direct or inverse correlations with IAT psychometric dimensions depending on gene and CpG site.	Included in qualitative synthesis and quantitative meta-analysis.
Annunzi et al. [23]	Structured conference abstract with extractable quantitative information.	Young adult university sample; detailed age and sex distribution were limited by the abstract format.	Internet Addiction Test/internet-addiction severity.	Salivary or peripheral DNA source described in abstract-level material; limited methodological detail.	OXTR.	Preliminary evidence of increased OXTR CpG4 methylation among participants with elevated IAT scores.	Included in qualitative synthesis and quantitative meta-analysis if the single effect remains in the final harmonized dataset; evaluated in sensitivity analysis restricted to full articles.
Marzi et al. [50]	Population-based longitudinal cohort/epigenome-wide study; full article.	Environmental Risk Longitudinal Twin study; 2232 twins born in England and Wales and assessed repeatedly from childhood to age 18; blood methylation available at age 18 after quality control.	Developmental victimization stress, including cybervictimization, and other forms of childhood/adolescent adversity.	Peripheral blood.	Epigenome-wide DNA methylation plus candidate stress-related genes, including NR3C1, FKBP5, BDNF, AVP, CRHR1, and SLC6A4.	Evidence for robust victimization-related DNA methylation differences was limited; candidate-gene analyses did not support predicted associations after additional controls.	Included in qualitative synthesis as a counterbalancing longitudinal/stress-exposure study; not included in pooled meta-analysis if no harmonizable digital-use effect was available.
Cerniglia et al. [47]	Exploratory cross-sectional study; full article.	Community youth sample from Rome, Italy; n = 71 (15 males, 56 females), median age 23 years (M = 22.67, SD = 0.359); analytic methylation subgroup included 32 participants with DAT1 10/10 genotype.	Internet Addiction Test, Barratt Impulsiveness Scale, and Adult Self-Report for emotional/behavioral problems.	Buccal swab/saliva DNA.	SLC6A3/DAT1 methylation loci 1–7.	Internet addiction was associated with impulsivity and behavioral/problem profiles; met5 and met7 methylation loci correlated with DAT methylation scores, but direct epigenetic prediction of psychopathological variables was preliminary.	Included in qualitative synthesis; not retained in pooled meta-analysis if effect-size information was not harmonizable.

**Table 4:** Synthesis of dimensions of finding, associated epigenetic mechanisms, and examples of results.

Dimension of Finding	Main Associated Epigenetic/ Biological Mechanism	Example of Key Finding (Baseline Study)
Problematic internet use/internet addiction	Differential DNA methylation in neurotransmission genes (OXTR, SLC6A3 (DAT1), SLC6A4 (SERT)), with a non-linear pattern according to the severity of the IAT.	Mild-risk users (30 < IAT < 49) showed significant methylation increases in OXTR (CpG2-4), SLC6A3 (DAT1; CpG1/CpG5) and inverse changes in SLC6A4 (SERT) compared to controls and to the IAT group > 50 (Annunzi et al. [22]).
Dopaminergic vulnerability, impulsivity and attachment	Correlations between SLC6A3 methylation loci (DAT1) and structural patterns of CpG covariation influenced by 3′-UTR genotype and relational/behavioral variables.	In a vulnerable youth sample, SLC6A3 (DAT1) methylation dynamics differentiated the subgroup with higher impulsivity and higher IAT scores versus securely attached controls, highlighting genetic and behavioral context-dependent CpG5-CpG6 and CpG3-CpG5 relationships [48].
Psychological and Behavioral Correlates of Problematic Internet Use	Association between methylation of specific sites of SLC6A3 (DAT1) and traits of impulsivity, rule-breaking, and behavioral withdrawal.	The met5 and met7 loci of SLC6A3 (DAT1) were positively correlated with internet addiction scores, and problematic internet use was further associated with impulsivity and behavioral problems in a sample of young people with SLC6A3 (DAT1) genotype 10/10 [47].
Circadian rhythmicity and problematic smartphone use	PER3 methylation as a biological moderator of the relationship between problematic mobile phone use and evening chronotype.	Problematic mobile phone use was associated with evening chronotype, and PER3 methylation showed a negative moderating effect on that association in young Chinese adults [24].
Victimization stress, including cybervictimization	Poor and poorly reproducible epigenetic signal in peripheral blood, both at the epigenomic level and in candidate genes related to stress.	In a population-based cohort, developmental victimization, including cybervictimization, showed very limited associations with DNA methylation, with no robust or replicable evidence after additional controls [50].
Preliminary epigenetic biomarkers in structured abstracts	Selective modulation of methylation in OXTR and SLC6A3 (DAT1) in preliminary studies with university samples.	A structured abstract showed increased methylation of OXTR CpG4 in students with IAT > 50, while another described methylation elevations in CpG5 and CpG7 of SLC6A3 (DAT1) in subjects with high IAT scores, which preliminarily broadens the base of candidate biomarkers (Annunzi et al. [23]; Annunzi et al. [49]).

Note. Although this section qualitatively summarizes the findings, four of the seven studies provided quantifiable effects that could be harmonized for quantitative synthesis. However, the evidence was marked by substantial heterogeneity in the type of effect reported, the biological tissue analyzed, and the function assigned to methylation (outcome, correlate, moderator, or structural pattern of covariation), so the interpretation of the meta-analysis should be carried out with caution and with special attention to the dependence between multiple effects from the same study [22,23,24,47,48,49].

## Data Availability

The original contributions presented in this study are included in the article/Supplementary Material. Further inquiries can be directed to the corresponding authors.

## Supplementary Materials

The following supporting information can be downloaded at https://www.mdpi.com/article/10.3390/epigenomes10030050/s1, Supplementary Material S1: Full data extraction table with harmonized effect sizes and imputation notes; Supplementary Material S2: Statistical software outputs and technical model diagnostics; Supplementary Material S3: Critical appraisal and risk-of-bias assessment of the included studies; Supplementary Material S4: Bootstrap contrasts across alternative ρ values by tissue type; Supplementary Material S5: Publication-bias assessment; Table S6: Reports of apparent eligibility excluded after assessment and primary reason.
