# Supplementary material for: DNA Methylation in Neurobiological Genes Associated with Problematic Digital Use in Adolescents and Young Adults: Systematic Review and Exploratory Multilevel Meta-Analysis of Emerging Evidence
Source: Epigenomes. 2026 Aug 3;10(3):50. doi: 10.3390/epigenomes10030050 (PMC13511636; doi:10.3390/epigenomes10030050)
Supplement: Supplementary file 1 [file epigenomes-10-00050-s001.zip › epigenomes-4399405-supplementary/SUPPLEMENTARY_MATERIAL_AJ_PDF/01_Supplementary_Materials_Cited_in_Manuscript/Supplementary Material S2. Statistical software outputs and technical model diagnostics.pdf]

Supplementary Material S2. Statistical software outputs and technical model diagnostics

DNA Methylation in Neurobiological Genes Associated with Problematic Digital Use in Adolescents and Young Adults: Systematic Review and Exploratory Multilevel Meta-Analysis of Emerging Evidence

Purpose. This supplementary file provides the statistical software outputs and technical diagnostic displays that support the multilevel meta-analysis, robust variance estimation, sensitivity analyses, moderator tests, Egger test, and tissue-type bootstrap contrasts summarized in the main manuscript. These outputs were removed from the main text to improve article readability and visual quality, while preserving analytical transparency and reproducibility in the Supplementary Materials.

Interpretation note. The screenshots below are technical outputs and should be interpreted together with the numerical summaries, methodological cautions, and sensitivity analyses reported in the manuscript. They are not intended to replace the main-text results but to document the statistical provenance of the reported estimates.

1. Main multilevel model and heterogeneity outputs

```
Multivariate Meta-Analysis Model (k = 41; method: REML)

  logLik  Deviance       AIC       BIC      AICc
-28.4078   56.8157   62.8157   67.8823   63.4823

Variance Components:

              estim    sqrt  nlvls  fixed       factor
sigma^2.1    0.0054  0.0736      4     no       author
sigma^2.2    0.1850  0.4302     41     no  author/es.id

Test for Heterogeneity:
Q(df = 40) = 435.9195, p-val < .0001

Model Results:

estimate      se    tval  df    pval   ci.lb   ci.ub
  0.2957  0.0896  3.2999  40  0.0020  0.1146  0.4769  **

---
Signif. codes:  0 ‘***’ 0.001 ‘**’ 0.01 ‘*’ 0.05 ‘.’ 0.1 ‘ ’ 1
```

Supplementary Figure S2.1. Pooled effect (meta-analysis). Effect size (Fisher’s z-transformed correlation) = 0.2957 (95% CI: 0.1146-0.4769). *(Former main-text Figure 2 before relocation to the Supplementary Materials.)*

```
        % of total variance    I2
Level 1            6.845641   ---
Level 2           90.505173 90.51
Level 3            2.649185  2.65
```

Supplementary Figure S2.2. Components of heterogeneity and sampling error. *(Former main-text Figure 3 before relocation to the Supplementary Materials.)*

2. Robust variance estimation and meta-regression outputs

```
Multivariate Meta-Analysis Model (k = 41; method: REML)

  logLik  Deviance       AIC       BIC      AICc
 -29.071    58.142    64.142    69.209    64.809

Variance Components:

           estim   sqrt  nlvls  fixed        factor
sigma^2.1  0.000  0.000      4     no        author
sigma^2.2  0.217  0.466     41     no  author/es.id

Test for Heterogeneity:
Q(df = 40) = 831.018, p-val < .001

Model Results:

estimate      se    zval    pval   ci.lb   ci.ub
   0.250  0.111  2.251  0.024  0.032  0.467   *

---
Signif. codes:  0 ‘***’ 0.001 ‘**’ 0.01 ‘*’ 0.05 ‘.’ 0.1 ‘ ’ 1
```

Supplementary Figure S2.3. Meta-analysis with robust variance estimation (RVE) of the association between problematic digital exposure and peripheral epigenetic alterations. *(Former main-text Figure 5 before relocation to the Supplementary Materials.)*

```
Multivariate Meta-Analysis Model (k = 41; method: REML)

  logLik  Deviance       AIC       BIC      AICc
   5.362   -10.724     7.276    21.014    14.776

Variance Components:

           estim   sqrt  nlvls  fixed        factor
sigma^2.1  0.000  0.000      4     no        author
sigma^2.2  0.005  0.070     41     no  author/es.id

Test for Residual Heterogeneity:
QE(df = 34) = 49.330, p-val = 0.043

Test of Moderators (coefficients 1:7):
F(df1 = 7, df2 = 34) = 47.284, p-val < .001

Model Results:

                                          estimate     se      tval  df    pval    ci.lb   ci.ub
EffectTypeBeta converted                    -0.078  0.049    -1.596  34   0.120  -0.176   0.021
EffectTypeCorrelation                        0.714  0.115     6.189  34   <.001   0.480   0.949  ***
EffectTypeGroupDiff                          0.511  0.040    12.662  34   <.001   0.429   0.593  ***
DirectionHypomethylation                    -1.009  0.092   -10.971  34   <.001  -1.195  -0.822  ***
DirectionNegative_methylation_association   -1.072  0.142    -7.553  34   <.001  -1.360  -0.784  ***
DirectionNegative_structural_coordination   -1.319  0.267    -4.942  34   <.001  -1.862  -0.777  ***
DirectionPositive_methylation_association   -0.241  0.129    -1.869  34   0.070  -0.503   0.021    .

---
Signif. codes:  0 ‘***’ 0.001 ‘**’ 0.01 ‘*’ 0.05 ‘.’ 0.1 ‘ ’ 1
```

Supplementary Figure S2.4. Meta-regression: high explained variability (total $R^2 \approx 97.4\%$). (*Former main-text Figure 6 before relocation to the Supplementary Materials.*)

3. Sensitivity analysis restricted to full-text articles

```
Multivariate Meta-Analysis Model (k = 40; method: REML)

  logLik  Deviance       AIC       BIC      AICc
-27.9159   55.8319   61.8319   66.8225   62.5176

Variance Components:

            estim    sqrt  nlvls  fixed        factor
sigma^2.1  0.0103  0.1015      3     no        author
sigma^2.2  0.1859  0.4312     40     no  author/es.id

Test for Heterogeneity:
Q(df = 39) = 432.3297, p-val < .0001

Model Results:

estimate      se    tval  df    pval   ci.lb   ci.ub
  0.2829  0.1023  2.7655  39  0.0086  0.0760  0.4899  **

---
Signif. codes:  0 '***' 0.001 '**' 0.01 '*' 0.05 '.' 0.1 ' ' 1
```

Supplementary Figure S2.5. Sensitivity meta-analysis restricted to full-text articles. The model excluded the only congress abstract effect included in the quantitative dataset, corresponding to Annunzi et al. [23], and retained 40 effect sizes. The pooled effect remained positive and statistically significant. *(Former main-text Figure 8 before relocation to the Supplementary Materials.)*

```
Multivariate Meta-Analysis Model (k = 40; method: REML)

  logLik  Deviance       AIC       BIC      AICc
  5.1432  -10.2865    7.7135   21.1821   15.5396

Variance Components:

            estim    sqrt  nlvls  fixed        factor
sigma^2.1  0.0134  0.1159      3     no        author
sigma^2.2  0.0051  0.0711     40     no  author/es.id

Test for Residual Heterogeneity:
QE(df = 33) = 48.9651, p-val = 0.0363

Test of Moderators (coefficients 1:7):
F(df1 = 7, df2 = 33) = 32.5713, p-val < .0001

Model Results:

                                      estimate      se      tval  df    pval    ci.lb    ci.ub
EffectTypeBeta converted               -0.0776  0.1260   -0.6156  33  0.5424  -0.3339   0.1788
EffectTypeCorrelation                   0.7144  0.1636    4.3652  33  0.0001   0.3814   1.0473  ***
EffectTypeGroupDiff                     0.5113  0.1228    4.1628  33  0.0002   0.2614   0.7611  ***
DirectionHypomethylation               -1.0086  0.0924  -10.9127  33  <.0001  -1.1966  -0.8206  ***
DirectionNegative_methylation_association  -1.0720  0.2171  -4.9387  33  <.0001  -1.5136  -0.6304  ***
DirectionNegative_structural_coordination  -1.3195  0.2673  -4.9370  33  <.0001  -1.8632  -0.7757  ***
DirectionPositive_methylation_association  -0.2476  0.2090  -1.1848  33  0.2446  -0.6727   0.1776

---
Signif. codes:  0 '***' 0.001 '**' 0.01 '*' 0.05 '.' 0.1 ' ' 1
```

Supplementary Figure S2.6. Meta-regression restricted to full-text articles. The model retained EffectType and Direction as moderators and showed a significant joint moderator structure, consistent with the main meta-regression. *(Former main-text Figure 9 before relocation to the Supplementary Materials.)*

```
Test of Moderators (coefficients 1:7):
F(df1 = 7, df2 = 33) = 32.5713, p-val < .0001
```

Supplementary Figure S2.7. Joint test of moderators for the sensitivity meta-regression restricted to full-text articles. The omnibus test remained statistically significant, supporting the contribution of the moderator structure. *(Former main-text Figure 10 before relocation to the Supplementary Materials.)*

```
> R2_total_aprox <- 1 - (sum(sigma2_con_mod) / sum(sigma2_sin_mod))
> cat("Varianza sin moderadores:", round(sum(sigma2_sin_mod), 4), "\n")
Varianza sin moderadores: 0.1962
> cat("Varianza con moderadores:", round(sum(sigma2_con_mod), 4), "\n")
Varianza con moderadores: 0.0185
> cat("R2 total aproximado:", round(R2_total_aprox, 4), "\n")
R2 total aproximado: 0.9057
```

Supplementary Figure S2.8. Approximate reduction of variance in the meta-regression restricted to full-text articles. The inclusion of moderators reduced total variance from 0.1962 to 0.0185, corresponding to an approximate R^2 of 0.9057. *(Former main-text Figure 11 before relocation to the Supplementary Materials.)*

4. Publication-bias and tissue-type moderation outputs

```
Test of Moderators (coefficient 2):'
F(df1 = 1, df2 = 2) = 11.3912, p-val = 0.0777
```

Supplementary Figure S2.9. Egger’s test.

```
         Test Adjustment CR_type Statistic    R p_val
1 CWB Adjusted       CR2     CR0   Naive-F 2000 0.3665
```

Supplementary Figure S2.10. Cluster wild bootstrap Wald test evaluating biological sample type (*Tissue*) as a moderator of epigenetic effect sizes under the main assumed intra-cluster correlation parameter ($\rho = 0.6$). The test used 2,000 bootstrap replications with CR2 variance correction and yielded a non-significant result (Naive-F test, $p = 0.3665$).

5. Influence diagnostics of the quantitative synthesis

Evaluation procedure

Influence diagnostics were performed on the main multilevel model estimated by REML, using effect sizes harmonized as Fisher's z-transformed correlations, their corresponding sampling variances, and a nested random-effects structure for effects within study/author (random = ~ 1 | author/es.id). In accordance with the methodological recommendations of Viechtbauer and Cheung [59] for identifying outliers and influential cases in meta-analysis, the assessment was conducted at two levels: (i) the individual-effect level, by successively deleting each of the 41 effect sizes; and (ii) the study/author cluster level, by successively deleting each of the 4 independent clusters. At both levels, Cook's distances and DFBETAS were calculated to assess whether any individual effect or study cluster disproportionately modified the pooled model estimate. These values were interpreted as exploratory sensitivity diagnostics, not as automatic criteria for exclusion.

Exploratory criteria for interpretation

For Cook's distances, the exploratory reference criterion was 4/n at the individual-effect level and 4/k at the cluster level, where n corresponds to the number of effect sizes and k to the number of study/author clusters. For DFBETAS, the corresponding criteria were 2/sqrt(n) and 2/sqrt(k). These cut-offs are heuristic and should be interpreted in light of the small number of independent studies, the dependence among effect sizes, and the substantive heterogeneity of the meta-analysis.

Table S2.1. Exploratory thresholds used in the influence diagnostics

Level of analysis	Number of units	Cook threshold	DFBETA threshold	Interpretive use
Individual effect	n = 41	4/n = 0.0976	2/sqrt(n) = 0.3123	Identify effects with relatively high influence
Study/author cluster	k = 4	4/k = 1.0000	2/sqrt(k) = 1.0000	Identify studies/clusters with relatively high influence

Results at the individual-effect level

At the individual-effect level, no effect size exceeded the exploratory Cook's distance threshold (4/n = 0.0976) or the maximum absolute DFBETA threshold (2/sqrt(n) = 0.3123). The highest Cook's distance corresponded to effect 8 from De Nardi et al. [44] (Cook = 0.0801; maximum absolute DFBETA = 0.1745), followed by other effects from the same study related to DAT1 covariation patterns. Secondary foci of relative influence were also observed in some effects from Annunzi et al. [23], mainly in estimates

associated with SERT. However, all values remained below the prespecified exploratory thresholds, indicating that no individual effect was identified as excessively influential.

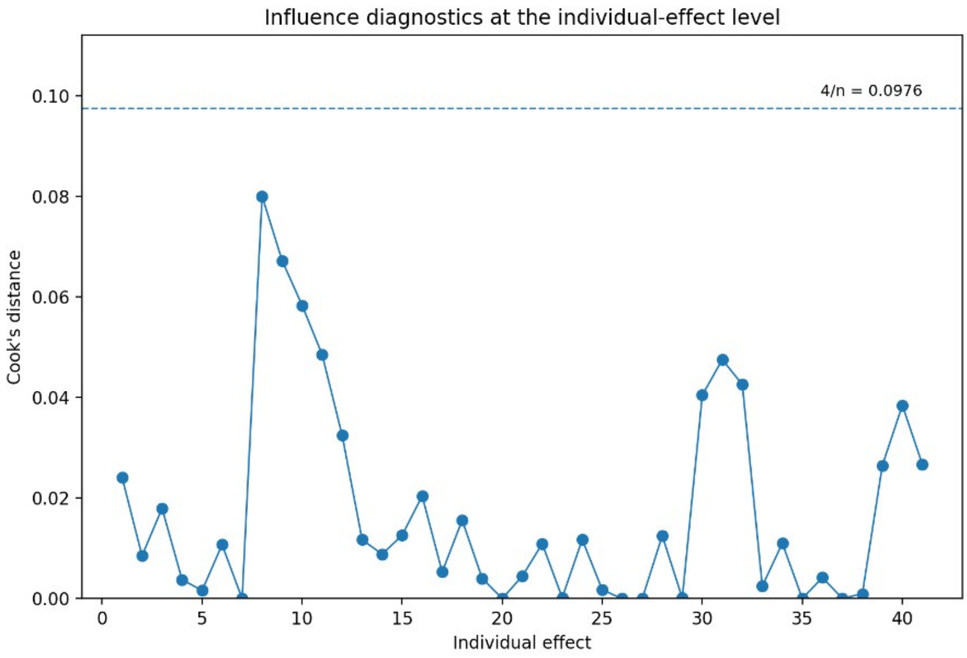

Supplementary Figure S2.11. Cook's distances for the 41 individual effect sizes. The dashed line represents the exploratory threshold 4/n = 0.0976.

Table S2.2. Individual effects with the highest Cook's distance

Effect	Study	Effect identifier	Cook	Max. abs. DFBETA	Exceeds threshold
8	De Nardi et al. (2020)	DeNardi2020_DAT1_M3M5_Control1010	0.0801	0.1745	No
9	De Nardi et al. (2020)	DeNardi2020_DAT1_M3M5_Control9x	0.0673	0.1637	No
10	De Nardi et al. (2020)	DeNardi2020_DAT1_M2M3_HighIAT	0.0584	0.2992	No
11	De Nardi et al. (2020)	DeNardi2020_DAT1_M2M3_Control1010	0.0486	0.2708	No
31	Annunzi et al. (2023)	Annunzi2023_SERT_CpG5_mild_vs_control	0.0476	0.2469	No
32	Annunzi et al. (2023)	Annunzi2023_SERT_CpG5_mild_vs_moderate	0.0426	0.2429	No
30	Annunzi et al.	Annunzi2023_SERT_CpG3_mild_vs_control	0.0405	0.2391	No

	(2023)				
40	Annunzi et al. (2023)	Annunzi2023_SERT_CpG3_spearman_IATlt50	0.0385	0.2346	No
12	De Nardi et al. (2020)	DeNardi2020_DAT1_M2M3_Control9x	0.0325	0.2193	No
41	Annunzi et al. (2023)	Annunzi2023_SERT_CpG5_spearman_IATlt50	0.0267	0.1973	No

Results at the study/author cluster level

At the study/author cluster level, no study exceeded the exploratory Cook's distance threshold (4/k = 1.0000) or the maximum absolute DFBETA threshold (2/sqrt(k) = 1.0000). The highest relative influence corresponded to De Nardi et al. [44] (Cook = 0.7843; maximum absolute DFBETA = 0.7695), followed by Li et al. [24] (Cook = 0.3017; maximum absolute DFBETA = 0.5472). In contrast, Annunzi et al. [23] and Annunzi et al. [22] showed low influence values at the cluster level. This pattern indicates differential contribution of studies to model fit, but it does not indicate excessive dependence of the pooled estimate on any single study under the exploratory criteria applied.

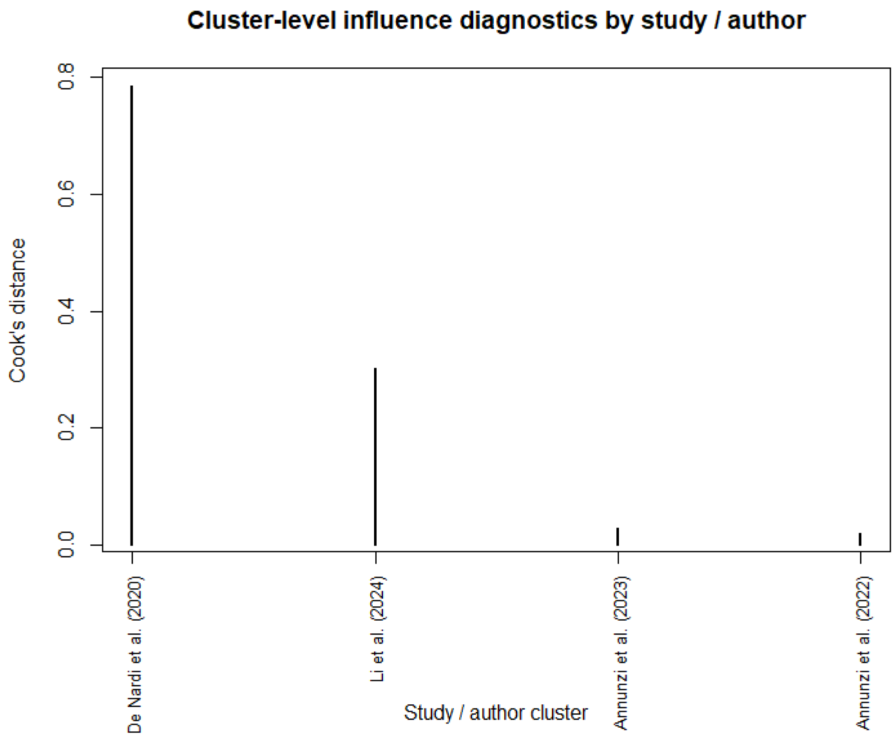

Supplementary Figure S2.12. Cook's distances at the study/author cluster level. The exploratory threshold 4/k = 1.0000 was not exceeded by any cluster.

Table S2.3. Influence diagnostics at the study/author cluster level

Study/cluster	n effects	Cook	Max. abs. DFBETA	Cook threshold 4/k	DFBETA threshold	Exceeds threshold
De Nardi et al. (2020)	12	0.7843	0.7695	1.0000	1.0000	No
Li et al. (2024)	3	0.3017	0.5472	1.0000	1.0000	No
Annunzi et al. (2023)	25	0.0288	0.0902	1.0000	1.0000	No
Annunzi et al. (2022)	1	0.0204	0.1276	1.0000	1.0000	No

Overall interpretation

The influence diagnostics support the overall stability of the pooled estimate from the multilevel meta-analysis. Although relative influence was not homogeneous, no individual effect or study/author cluster

exceeded the exploratory Cook's distance or DFBETA thresholds. The higher relative influence of De Nardi et al. [44] is consistent with its contribution of multiple effects derived from DAT1 covariation patterns and with the internal variability observed across effects. The secondary influence of Li et al. [24] should be interpreted in relation to its distinct methodological role, because it modelled PER3 methylation as a biological moderator of the association between problematic mobile phone use and chronotype. The individual foci observed in effects from Annunzi et al. [23] were mainly concentrated in SERT-related estimates, but they did not reach magnitudes sufficient to be considered influential under the prespecified criteria.

Taken together, these results indicate that the positive pooled effect reported in the manuscript was not driven by a single study or by a single effect size. Therefore, the influence diagnostics are consistent with the general interpretation of the manuscript: there is a positive and biologically plausible global association between problematic digital exposure and peripheral DNA methylation differences, but the evidence remains preliminary and methodologically heterogeneous.
