# Supplementary material for: DNA Methylation in Neurobiological Genes Associated with Problematic Digital Use in Adolescents and Young Adults: Systematic Review and Exploratory Multilevel Meta-Analysis of Emerging Evidence
Source: Epigenomes. 2026 Aug 3;10(3):50. doi: 10.3390/epigenomes10030050 (PMC13511636; doi:10.3390/epigenomes10030050)
Supplement: Supplementary file 1 [file epigenomes-10-00050-s001.zip › epigenomes-4399405-supplementary/SUPPLEMENTARY_MATERIAL_AJ_PDF/01_Supplementary_Materials_Cited_in_Manuscript/Supplementary Material S3. Critical appraisal and risk-of-bias assessment of the included studies.pdf]

Supplementary Material S3. Critical appraisal and risk-of-bias assessment of the included studies

Note: Study citations are synchronized with the MDPI numerical citation format used in the manuscript.

Assessment procedure

The critical appraisal and risk-of-bias assessment were adapted to the design and publication format of each included study. Cross-sectional studies published as full-text articles were assessed using AXIS, because this tool is specifically designed for observational cross-sectional studies and allows structured appraisal of objectives, design, sampling, measurement, statistical methods, reporting quality, and interpretation. The longitudinal cohort study by Marzi et al. [46] was assessed using ROBINS-E, given its observational exposure-outcome structure and its aim of estimating the association between victimization and DNA methylation. The structured conference abstracts by Annunzi et al. [23,45] were assessed using AACODS, because they represent grey literature with abbreviated methodological reporting and because this tool allows appraisal of authority, accuracy, coverage, objectivity, date, and significance. For genetic and epigenetic studies, STROBE/STREGA criteria were considered as supporting criteria to appraise reporting transparency, but they did not replace the main appraisal tool assigned according to study design.

Table S3.1. Individual assessment by study

Study (MDPI ref.)	Design	Tool	Risk of bias	CodeROB	Quality	CodeQuality	Main reason for risk-of-bias judgment
Annunzi et al. [22]	Cross-sectional; full-text article; university sample; saliva	AXIS; STROBE/STREGA support	Moderate	2	Moderate	2	Clear biological rationale and explicit molecular measurement, but modest university-based sample, candidate-gene approach, self-reported IAT exposure, subgroup comparisons, limited confounding control, and no causal inference.
Annunzi et al. [23]	Structured conference abstract; university sample; saliva	AACODS	Moderate	2	Moderate	2	Preliminary grey-literature evidence with identifiable sample, IAT exposure, salivary DNA methylation, and quantitative results; limited by abbreviated reporting and incomplete information on confounding control, multiplicity, statistical power, and reproducibility.

Study (MDPI ref.)	Design	Tool	Risk of bias	CodeROB	Quality	CodeQuality	Main reason for risk-of-bias judgment
Li et al. [24]	Cross-sectional; full-text article; young adults; peripheral blood	AXIS	Moderate	2	High	3	Large sample size, blood-based methylation measurement, and multivariable analyses strengthen the study; however, the design is cross-sectional, problematic mobile phone use and chronotype were self-reported, and residual confounding or reverse causality cannot be excluded.
Cerniglia et al. [43]	Cross-sectional; full-text article; restricted genotype group; buccal swab	AXIS; STROBE/STREGA support	Moderate	2	Moderate	2	Full-text article with explicit instruments and molecular measurement; limited by very small and restricted sample, exploratory correlational design, low statistical power, incomplete confounding control, and absence of temporal inference.
De Nardi et al. [44]	Cross-sectional; full-text article; community/youth sample; buccal swab	AXIS; STROBE/STREGA support	Moderate	2	Moderate	2	Article with clear psychometric and molecular procedures; limited by a posteriori subgroup selection, small analytic subgroups, non-probabilistic recruitment, limited confounding control, and absence of causal inference.
Annunzi et al. [45]	Structured conference abstract; university sample; saliva/salivary exosomes	AACODS	Moderate	2	Moderate	2	Preliminary conference evidence with sample, IAT, saliva, methylation/genetic targets, and quantitative signal; limited by abbreviated methods, incomplete detail on sampling, bias control, statistical transparency, and independent reproducibility.
Marzi et al. [46]	Population-based longitudinal cohort; peripheral blood	ROBINS-E	Low to moderate	2	High	3	Strong longitudinal cohort, large sample, prospective victimization assessment, EWAS, smoking adjustment, and co-twin control analyses; remaining concerns relate to residual confounding, peripheral tissue specificity, and indirectness regarding problematic digital exposure.

Coding scheme: risk of bias: low = 1, moderate = 2, high = 3. Methodological quality: low = 1, moderate = 2, high = 3.

Overall risk-of-bias assessment

The overall body of evidence was classified as having moderate risk of bias. This judgment reflects the predominance of observational and exploratory designs, frequent use of self-reported digital exposure measures, non-probabilistic or university-based samples, small biological subsamples, incomplete confounding control, heterogeneity in peripheral tissues and epigenetic targets, and limited capacity for temporal or causal inference. Nevertheless, no study was judged to have high or critical risk of bias when bias itself was distinguished from imprecision, indirectness,

or reporting limitations. The two structured conference abstracts were not automatically penalized as high risk because of their publication format; they were classified as moderate risk with limited methodological transparency and interpreted as preliminary hypothesis-generating evidence. Overall, the evidence supports a biologically plausible association between problematic digital exposure or related digital stressors and peripheral DNA methylation differences.

However, the certainty of the evidence remains limited by methodological heterogeneity, the small number of independent studies, the exploratory nature of several analyses, and the limited availability of longitudinal or independently replicated evidence. Therefore, the findings should be interpreted as preliminary and hypothesis-generating rather than confirmatory or directly applicable for individual diagnostic or prognostic purposes.
