# Supplementary material for: DNA Methylation in Neurobiological Genes Associated with Problematic Digital Use in Adolescents and Young Adults: Systematic Review and Exploratory Multilevel Meta-Analysis of Emerging Evidence
Source: Epigenomes. 2026 Aug 3;10(3):50. doi: 10.3390/epigenomes10030050 (PMC13511636; doi:10.3390/epigenomes10030050)
Supplement: Supplementary file 1 [file epigenomes-10-00050-s001.zip › epigenomes-4399405-supplementary/SUPPLEMENTARY_MATERIAL_AJ_PDF/01_Supplementary_Materials_Cited_in_Manuscript/Supplementary Material S4. Bootstrap contrasts across alternative rho values by tissue type.pdf]

Supplementary Analysis S4. Sensitivity analysis for tissue type as moderator

As a sensitivity analysis for the hypothesis test of biological sample type (*Tissue*) as a moderator of epigenetic effect sizes, a cluster-robust Wald test using *cluster wild bootstrap* (CWB), CR2 variance correction, and 2,000 bootstrap replications was performed. The analysis evaluated a broad range of assumptions for the intra-cluster correlation parameter: low correlation ($\rho = 0.1$), moderate correlation ($\rho = 0.3$–0.5), high correlation ($\rho = 0.6$), and very high correlation ($\rho = 0.9$). The null hypothesis (H_0) stated that biological sample type does not moderate epigenetic effect sizes, whereas the alternative or research hypothesis (H_1) stated that biological sample type has a statistically significant moderating effect. The bootstrap graphical outputs and the corresponding *Test adjustment: CWB* results are presented in Supplementary Figures S4.1–S4.5: S4.1 for $\rho = 0.1$, S4.2 for $\rho = 0.3$, S4.3 for $\rho = 0.6$, S4.4 for $\rho = 0.9$, and S4.5 for $\rho = 0.5$. Across all evaluated scenarios, the p-values remained above the conventional threshold for statistical significance ($\rho = 0.1$, $p = 0.2475$; $\rho = 0.3$, $p = 0.1220$; $\rho = 0.5$, $p = 0.1785$; $\rho = 0.6$, $p = 0.3665$; $\rho = 0.9$, $p = 0.4170$), indicating that the inferential decision was stable across wide variations in intra-cluster correlation assumptions. Therefore, H_0 was not rejected. These findings suggest that, in this exploratory dataset, differences among saliva, buccal swab, and peripheral blood did not statistically explain the variability in epigenetic effect sizes. This result supports one of the main interpretations of the present study: the overall association between problematic digital exposure and peripheral DNA methylation differences does not appear to depend, in a statistically demonstrable way, on the biological sample type analyzed. Nevertheless, given the high heterogeneity, the limited number of independent studies, and the emerging nature of the available evidence, this finding should be interpreted as an absence of statistical evidence for tissue-type moderation in the present study, rather than as definitive evidence of biological equivalence across tissues (see Supplementary Figures S4.1–S4.5).

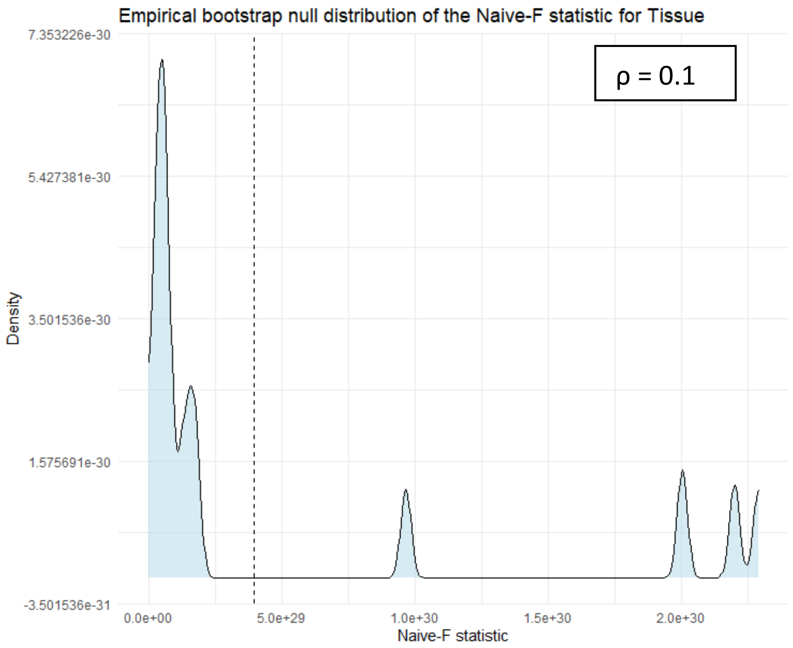

	Test	Adjustment	CR_type	Statistic	R	p_val
CWB	Adjusted	CR2	CR0	Naive-F	2000	0.2475

Figure S4.1

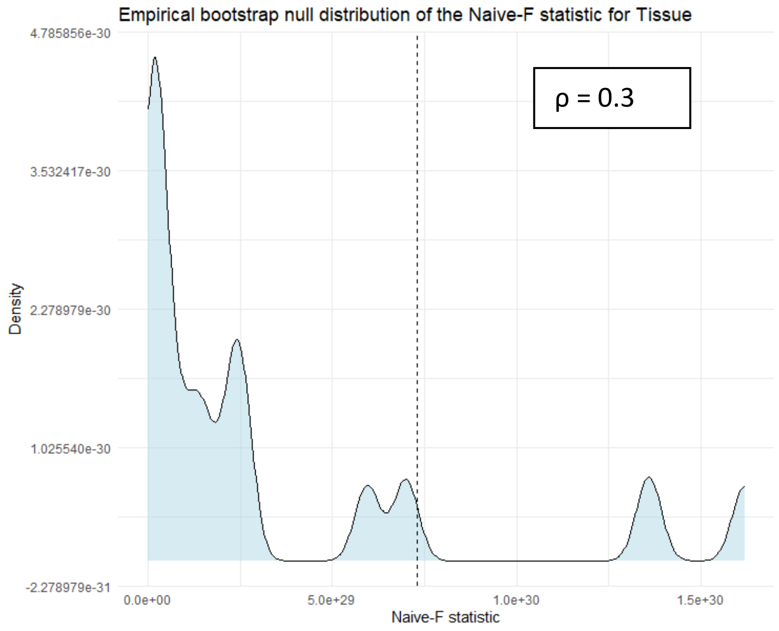

	Test	Adjustment	CR_type	Statistic	R	p_val
CWB	Adjusted	CR2	CR0	Naive-F	2000	0.122

Figure S4.2

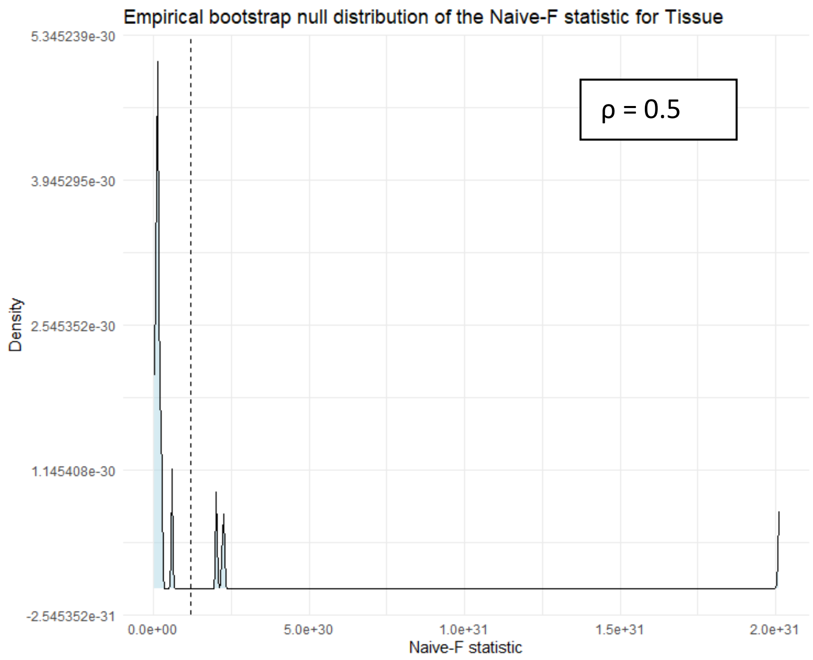

	Test	Adjustment	CR_type	Statistic	R	p_val
CWB	Adjusted	CR2	CR0	Naive-F	2000	0.1785

Figure S4.3

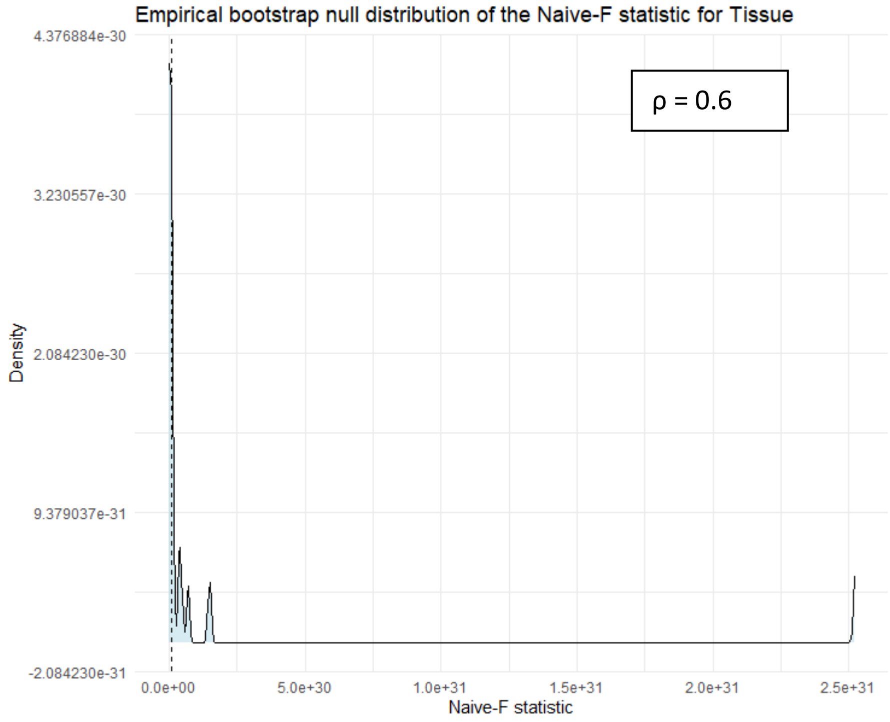

	Test	Adjustment	CR_type	Statistic	R	p_val
CWB	Adjusted	CR2	CR0	Naive-F	2000	0.3665

Figure S4.4

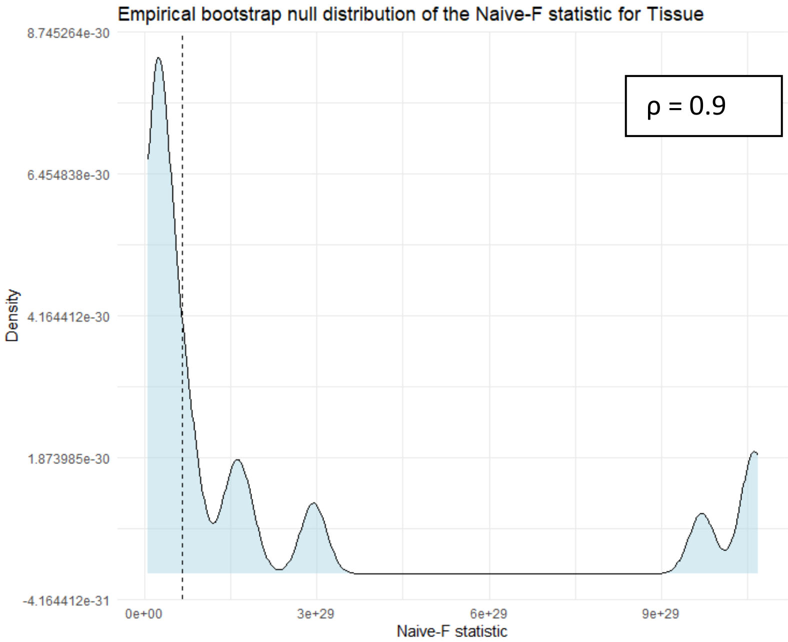


```
       Test Adjustment CR_type Statistic    R p_val
CWB Adjusted        CR2     CR0   Naive-F 2000 0.417
```

Figure S4.5
