# Supplementary material for: DNA Methylation in Neurobiological Genes Associated with Problematic Digital Use in Adolescents and Young Adults: Systematic Review and Exploratory Multilevel Meta-Analysis of Emerging Evidence
Source: Epigenomes. 2026 Aug 3;10(3):50. doi: 10.3390/epigenomes10030050 (PMC13511636; doi:10.3390/epigenomes10030050)
Supplement: Supplementary file 1 [file epigenomes-10-00050-s001.zip › epigenomes-4399405-supplementary/SUPPLEMENTARY_MATERIAL_AJ_PDF/01_Supplementary_Materials_Cited_in_Manuscript/Supplementary Material S5. Publication-bias assessment.pdf]

Supplementary Material S5. Evaluation of publication bias in the quantitative synthesis

Evaluation procedure

The risk of publication bias in the quantitative synthesis was evaluated through visual inspection of the funnel plot and Egger's regression test, in accordance with methodological recommendations for meta-analysis and for the interpretation of funnel-plot asymmetry [62-65]. The analysis was planned as a complementary and exploratory assessment because of the multilevel structure of the meta-analysis, the dependence among effect sizes derived from the same studies, and the small number of independent studies. The following hypotheses were tested: H0, no evidence of publication bias or small-study effects (intercept = 0); H1, possible publication bias or small-study effects (intercept different from 0). The prespecified criterion was to reject H0 if $p < 0.05$.

Graphical and statistical results

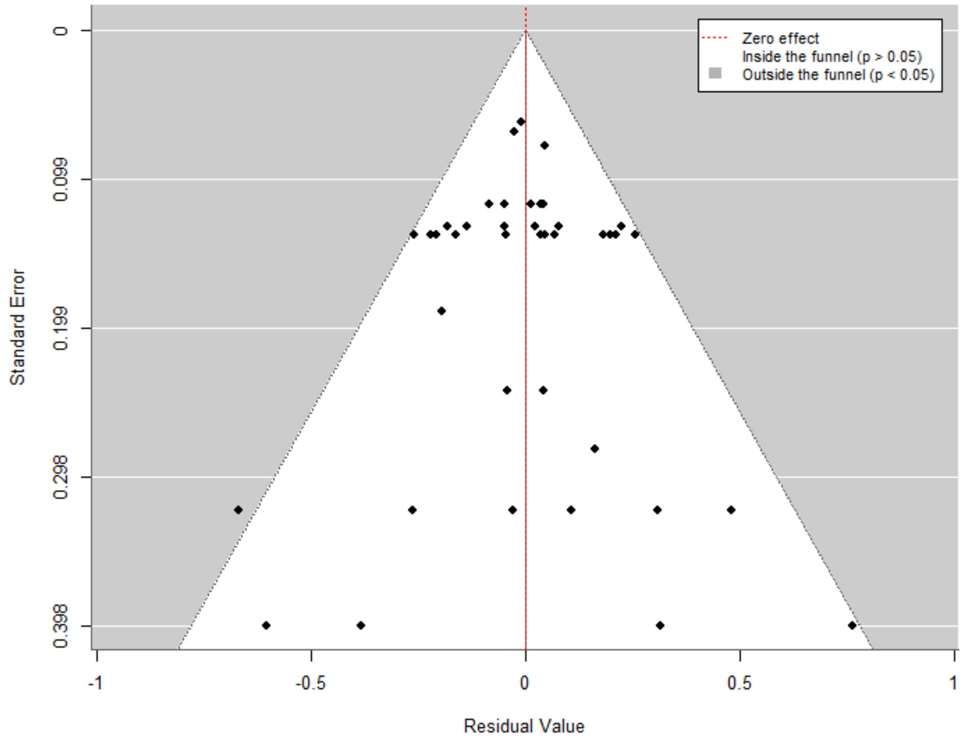

Figure 12. Funnel plot.

Figure S5.1. Funnel plot for the visual assessment of publication bias. The red vertical line represents the null effect; the white area corresponds to the expected funnel for results within the threshold of statistical non-significance, and the grey areas represent zones outside the funnel.

```
Test of Moderators (coefficient 2):'
F(df1 = 1, df2 = 2) = 11.3912, p-val = 0.0777
```

Figure 13. Egger's Test.

Figure S5.2. Egger's regression test for the statistical assessment of funnel-plot asymmetry. Result: F = 11.3912 (df1 = 1; df2 = 2), with p-value = 0.0777.

Table S5.1. Summary of the publication-bias assessment

Component	Observed result	Interpretation and methodological judgment
Funnel plot	Overall distribution around the vertical null-effect line, with dispersion at the base and some points outside the expected funnel.	No extreme asymmetry or unequivocal pattern of systematic absence of studies was observed. The basal dispersion may reflect low precision and heterogeneity. Judgment: no conclusive visual evidence of publication bias.
Egger's test	F = 11.3912 (df1 = 1; df2 = 2), p-value = 0.0777.	The p-value is greater than 0.05; therefore, H0 was not rejected. The test should be considered exploratory because of the small number of independent units and the dependence among effect sizes.
Consistency between methods	The visual inspection and the statistical test did not show conclusive evidence of asymmetry.	Both results point to insufficient evidence to affirm publication bias; however, they do not allow publication bias to be definitively ruled out. Judgment: non-conclusive risk, probably low to moderate.
Limitations of the assessment	Small number of independent studies, dependence among effects, high heterogeneity, and methodological variation.	These conditions reduce the diagnostic capacity of the funnel plot and Egger's test. The assessment should be interpreted as complementary rather than confirmatory.

Overall, the assessment of publication bias using the funnel plot and Egger's test did not provide statistically conclusive evidence of publication bias in the quantitative synthesis. Egger's test was not significant (p-value = 0.0777), and the funnel plot did not show an extreme or unequivocal pattern of asymmetry. Nevertheless, because of the small number of independent studies, the dependence among effect sizes, and the substantive heterogeneity of the evidence, this assessment should be interpreted as exploratory.
