# Supplementary material for: DNA Methylation in Neurobiological Genes Associated with Problematic Digital Use in Adolescents and Young Adults: Systematic Review and Exploratory Multilevel Meta-Analysis of Emerging Evidence
Source: Epigenomes. 2026 Aug 3;10(3):50. doi: 10.3390/epigenomes10030050 (PMC13511636; doi:10.3390/epigenomes10030050)
Supplement: Supplementary file 1 [file epigenomes-10-00050-s001.zip › epigenomes-4399405-supplementary/SUPPLEMENTARY_MATERIAL_AJ_PDF/01_Supplementary_Materials_Cited_in_Manuscript/Table S6. Reports of apparent eligibility excluded after assessment and primary reason.pdf]

SUPPLEMENTARY TABLE S6

Reports of apparent eligibility excluded after assessment and primary reason

The PRISMA flow diagram presents the aggregate reasons for all 353 exclusions after eligibility assessment. This table cites the reports identified during the audit that could reasonably be mistaken for eligible studies because their titles or abstracts refer to digital exposure, epigenetic or DNA methylation outcomes, youth, or social networks.

No.	Report	Year	DOI / identifier	Primary reason for exclusion	Eligibility criterion not met
1	Batool ST, Ali U, Mahato RK. Adolescent social-media exposure and cortical thinning signals: latest 2025 MRI-epigenome correlations.	2026	10.1097/MS9.0000000000004645	Letter to the editor; it does not report an original primary study with extractable participant-level or effect-size data.	Ineligible publication type and absence of original empirical data.
2	Malik S, Chourasia A, Pradhan RK. Prolonged Effects of Radiofrequency Electromagnetic Radiation Emanated from Mobile Phone on Global DNA Methylation and Spawning in Zebrafish, Danio rerio.	2021	Scopus record; no DOI identified in the audited workbook	Experimental study conducted in zebrafish rather than human participants.	Non-human population.
3	Immunoepigenetic-gut microbiome axis in the social networks of health disparate youth.	2019	Project/funding record; no article DOI identified	Project record rather than a report of original study results; “social networks” refers to community/interpersonal networks rather than digital social-media exposure.	Ineligible report type and no relevant digital exposure.

Note. The remaining exclusions are summarized in the flow diagram: 349 reports without a relevant problematic or stressful digital exposure linked to epigenetic or DNA methylation outcomes, two non-human reports, one letter to the editor, and one abstract with insufficient data. Table S6 focuses on reports that could reasonably appear eligible, as required by PRISMA 2020 item 16b.
