# Supplementary material for: DNA Methylation in Neurobiological Genes Associated with Problematic Digital Use in Adolescents and Young Adults: Systematic Review and Exploratory Multilevel Meta-Analysis of Emerging Evidence
Source: Epigenomes. 2026 Aug 3;10(3):50. doi: 10.3390/epigenomes10030050 (PMC13511636; doi:10.3390/epigenomes10030050)
Supplement: Supplementary file 1 [file epigenomes-10-00050-s001.zip › epigenomes-4399405-supplementary/SUPPLEMENTARY_MATERIAL_AJ_PDF/02_Editorial_PRISMA_Documents/PRISMA 2020 Checklist.pdf]

PRISMA 2020 Checklist

Section and Topic	Item #	Checklist item	Location where item is reported
**TITLE**			
Title	1	Identify the report as a systematic review.	Submitted manuscript, p. 1, title.
**ABSTRACT**			
Abstract	2	See the PRISMA 2020 for Abstracts checklist.	Submitted manuscript, p. 1, structured abstract.
**INTRODUCTION**			
Rationale	3	Describe the rationale for the review in the context of existing knowledge.	Submitted manuscript, pp. 2-3, Introduction.
Objectives	4	Provide an explicit statement of the objective(s) or question(s) the review addresses.	Submitted manuscript, pp. 3-4, review question and Section 2 (Objectives).
**METHODS**			
Eligibility criteria	5	Specify the inclusion and exclusion criteria for the review and how studies were grouped for the syntheses.	Submitted manuscript, pp. 7-8, Table 2 and eligibility text; p. 9, criteria for inclusion in the quantitative synthesis.
Information sources	6	Specify all databases, registers, websites, organisations, reference lists and other sources searched or consulted to identify studies. Specify the date when each source was last searched or consulted.	Submitted manuscript, pp. 5-8, Section 3.1 and Table 1; Supplementary PRISMA 2020 Reporting Addendum, Item 6 (last search in all databases: 11 June 2026; no additional eligible studies identified).
Search strategy	7	Present the full search strategies for all databases, registers and websites, including any filters and limits used.	Submitted manuscript, pp. 5-8, Table 1 (full database-specific search strategies, filters, and limits).
Selection process	8	Specify the methods used to decide whether a study met the inclusion criteria of the review, including how many reviewers screened each record and each report retrieved, whether they worked independently, and if applicable, details of automation tools used in the process.	Submitted manuscript, p. 8, Section 3.1 and Figure 1; Supplementary PRISMA 2020 Reporting Addendum, Item 8.
Data collection process	9	Specify the methods used to collect data from reports, including how many reviewers collected data from each report, whether they worked independently, any processes for obtaining or confirming data from study investigators, and if applicable, details of automation tools used in the process.	Submitted manuscript, p. 9, Section 3.2; Supplementary Material 1; Supplementary PRISMA 2020 Reporting Addendum, Item 9.
Data items	10a	List and define all outcomes for which data were sought. Specify whether all results that were compatible with each outcome domain in each study were sought (e.g. for all measures, time points, analyses), and if not, the methods used to decide which results to collect.	Submitted manuscript, p. 9, Section 3.2; Supplementary Material 1 (reported effects, harmonized correlations, Fisher z values, standard errors/variances, effect type/domain, direction, and primary-analysis inclusion).
	10b	List and define all other variables for which data were sought (e.g. participant and intervention characteristics, funding sources). Describe any assumptions made about any missing or unclear information.	Submitted manuscript, pp. 9-10; Supplementary Material 1 (gene/CpG site, exposure, tissue, age, country, sample size, study design, and inference notes). Assumptions for incomplete statistics are described on p. 9 and documented in Supplementary Material 1.
Study risk of bias assessment	11	Specify the methods used to assess risk of bias in the included studies, including details of the tool(s) used, how many reviewers assessed each study and whether they worked independently, and if applicable, details of automation tools used in the process.	Submitted manuscript, p. 19, Section 4.6; Supplementary Material 3; Supplementary PRISMA 2020 Reporting Addendum, Item 11.
Effect measures	12	Specify for each outcome the effect measure(s) (e.g. risk ratio, mean difference) used in the synthesis or presentation of results.	Submitted manuscript, p. 9, Section 3.2 (correlations transformed to Fisher z); effect estimates and precision reported in the quantitative-results section and Supplementary Material 1.
Synthesis methods	13a	Describe the processes used to decide which studies were eligible for each synthesis (e.g. tabulating the study intervention characteristics and comparing against the planned groups for each synthesis (item #5)).	Submitted manuscript, p. 9, Section 3.2; Supplementary Material 1 (PrimaryAnalysisInclude field).
	13b	Describe any methods required to prepare the data for presentation or synthesis, such as handling of missing summary statistics, or data conversions.	Submitted manuscript, p. 9, Section 3.2 (effect-size harmonization and conservative prespecified imputations); Supplementary Material 1 (inference flags and notes).
	13c	Describe any methods used to tabulate or visually display results of individual studies and syntheses.	Submitted manuscript, Results section, Tables 3-4 and Figures 2-13; Supplementary Materials 1-5.

Section and Topic	Item #	Checklist item	Location where item is reported
	13d	Describe any methods used to synthesize results and provide a rationale for the choice(s). If meta-analysis was performed, describe the model(s), method(s) to identify the presence and extent of statistical heterogeneity, and software package(s) used.	Submitted manuscript, p. 9, Section 3.2, and quantitative-results section (multilevel random-effects models, REML, heterogeneity decomposition, R/RStudio and meta-analytic packages).
	13e	Describe any methods used to explore possible causes of heterogeneity among study results (e.g. subgroup analysis, meta-regression).	Submitted manuscript, meta-regression and moderator-analysis sections.
	13f	Describe any sensitivity analyses conducted to assess robustness of the synthesized results.	Submitted manuscript, Section 3.2 and sensitivity-analysis sections (robust variance estimation, restriction to full articles, influence diagnostics, and bootstrap tissue analysis); Supplementary Materials 2 and 4.
Reporting bias assessment	14	Describe any methods used to assess risk of bias due to missing results in a synthesis (arising from reporting biases).	Submitted manuscript, Section 4.9 (funnel plot and Egger regression test); Supplementary Material 5.
Certainty assessment	15	Describe any methods used to assess certainty (or confidence) in the body of evidence for an outcome.	Supplementary PRISMA 2020 Reporting Addendum, Item 15. No formal GRADE or other structured certainty assessment was performed; confidence was appraised narratively.
**RESULTS**			
Study selection	16a	Describe the results of the search and selection process, from the number of records identified in the search to the number of studies included in the review, ideally using a flow diagram.	Submitted manuscript, p. 8, Figure 1 (PRISMA 2020 flow diagram) and accompanying selection results.
	16b	Cite studies that might appear to meet the inclusion criteria, but which were excluded, and explain why they were excluded.	Submitted manuscript, p. 8, Figure 1; Supplementary PRISMA 2020 Reporting Addendum, Item 16b; Supplementary Table S6, “Reports of apparent eligibility excluded after assessment and primary reason.” Of 360 reports assessed for eligibility, 353 were excluded: 349 lacked a relevant problematic or stressful digital exposure linked to epigenetic or DNA methylation outcomes, two were non-human studies, one was a letter to the editor, and one was an abstract with insufficient data.
Study characteristics	17	Cite each included study and present its characteristics.	Submitted manuscript, Sections 4.1-4.5, Tables 3-4; Supplementary Material 1.
Risk of bias in studies	18	Present assessments of risk of bias for each included study.	Submitted manuscript, p. 19, Section 4.6; Supplementary Material 3, study-level assessments.
Results of individual studies	19	For all outcomes, present, for each study: (a) summary statistics for each group (where appropriate) and (b) an effect estimate and its precision (e.g. confidence/credible interval), ideally using structured tables or plots.	Submitted manuscript, qualitative results by study, forest plot/quantitative-results section, and Supplementary Material 1 (individual effect-size data).
Results of syntheses	20a	For each synthesis, briefly summarise the characteristics and risk of bias among contributing studies.	Submitted manuscript, Sections 4.6-4.7; Supplementary Material 3.
	20b	Present results of all statistical syntheses conducted. If meta-analysis was done, present for each the summary estimate and its precision (e.g. confidence/credible interval) and measures of statistical heterogeneity. If comparing groups, describe the direction of the effect.	Submitted manuscript, quantitative-results sections, Figures 2-13.
	20c	Present results of all investigations of possible causes of heterogeneity among study results.	Submitted manuscript, meta-regression and moderator-analysis sections.
	20d	Present results of all sensitivity analyses conducted to assess the robustness of the synthesized results.	Submitted manuscript, sensitivity-analysis sections; Supplementary Materials 2 and 4.
Reporting biases	21	Present assessments of risk of bias due to missing results (arising from reporting biases) for each synthesis assessed.	Submitted manuscript, Section 4.9; Supplementary Material 5.
Certainty of evidence	22	Present assessments of certainty (or confidence) in the body of evidence for each outcome assessed.	Submitted manuscript, Discussion and Conclusions (narrative appraisal); Supplementary PRISMA 2020 Reporting Addendum, Item 22. No formal outcome-level certainty ratings were produced.
**DISCUSSION**			
Discussion	23a	Provide a general interpretation of the results in the context of other evidence.	Submitted manuscript, Section 5, Discussion.
	23b	Discuss any limitations of the evidence included in the review.	Submitted manuscript, Section 5, Discussion (limitations of the included evidence).

PRISMA

PRISMA 2020 Checklist

Section and Topic	Item #	Checklist item	Location where item is reported
	23c	Discuss any limitations of the review processes used.	Submitted manuscript, Section 5, Discussion (limitations of the review and quantitative-synthesis processes).
	23d	Discuss implications of the results for practice, policy, and future research.	Submitted manuscript, Sections 5-7, Discussion, Conclusions, and Recommendations.
**OTHER INFORMATION**			
Registration and protocol	24a	Provide registration information for the review, including register name and registration number, or state that the review was not registered.	Submitted manuscript, p. 5: PROSPERO registration CRD420261431295.
	24b	Indicate where the review protocol can be accessed, or state that a protocol was not prepared.	Submitted manuscript, p. 5; Supplementary PRISMA 2020 Reporting Addendum, Item 24b. Protocol: https://www.crd.york.ac.uk/PROSPERO/view/CRD420261431295.
	24c	Describe and explain any amendments to information provided at registration or in the protocol.	Supplementary PRISMA 2020 Reporting Addendum, Item 24c. No protocol amendments were reported by the review team.
Support	25	Describe sources of financial or non-financial support for the review, and the role of the funders or sponsors in the review.	Submitted manuscript, Funding statement; Supplementary PRISMA 2020 Reporting Addendum, Item 25 (publication costs funded by Universidad Santiago de Cali; funder role specified).
Competing interests	26	Declare any competing interests of review authors.	Submitted manuscript, Conflicts of Interest Statement; Supplementary PRISMA 2020 Reporting Addendum, Item 26.
Availability of data, code and other materials	27	Report which of the following are publicly available and where they can be found: template data collection forms; data extracted from included studies; data used for all analyses; analytic code; any other materials used in the review.	Submitted manuscript, Supplementary Materials statement; Supplementary Materials 1-5; Supplementary PRISMA 2020 Reporting Addendum, Item 27 (complete analysis dataset and R code availability).

Reporting note: This checklist is completed against the submitted manuscript, its five supplementary files, and the accompanying Supplementary PRISMA 2020 Reporting Addendum.

From: Page MJ, McKenzie JE, Bossuyt PM, Boutron I, Hoffmann TC, Mulrow CD, et al. The PRISMA 2020 statement: an updated guideline for reporting systematic reviews. BMJ 2021;372:n71. doi: 10.1136/bmj.n71. This work is licensed under CC BY 4.0. To view a copy of this license, visit https://creativecommons.org/licenses/by/4.0/
