# Supplementary material for: DNA Methylation in Neurobiological Genes Associated with Problematic Digital Use in Adolescents and Young Adults: Systematic Review and Exploratory Multilevel Meta-Analysis of Emerging Evidence
Source: Epigenomes. 2026 Aug 3;10(3):50. doi: 10.3390/epigenomes10030050 (PMC13511636; doi:10.3390/epigenomes10030050)
Supplement: Supplementary file 1 [file epigenomes-10-00050-s001.zip › epigenomes-4399405-supplementary/SUPPLEMENTARY_MATERIAL_AJ_PDF/02_Editorial_PRISMA_Documents/SUPPLEMENTARY_PRISMA_2020.pdf]

SUPPLEMENTARY PRISMA 2020 REPORTING ADDENDUM

DNA Methylation in Neurobiological Genes Associated with Problematic Digital Use in Adolescents and Young Adults: Systematic Review and Exploratory Multilevel Meta-Analysis of Emerging Evidence

Purpose. This addendum provides PRISMA 2020 reporting details that are not fully explicit in the submitted manuscript. It supplements reporting only and does not modify the eligibility decisions, extracted data, statistical analyses, results, or conclusions of the review.

PRISMA item	Topic	Supplementary reporting information
**6**	**Information sources and search update**	Scopus, PubMed/MEDLINE, Web of Science, Dimensions, Lens, and ScienceDirect were searched. The last search in all six sources was conducted on 11 June 2026. This update did not identify any additional eligible studies and therefore did not change the body of evidence included in the systematic review or meta-analysis.
**8**	**Selection process**	Two reviewers independently screened every title and abstract and subsequently assessed every potentially eligible full-text report against the predefined eligibility criteria. Disagreements were first discussed to reach consensus; unresolved disagreements were adjudicated by the third author. Database-native filters, including publication year and document type, were applied as part of the search process. Microsoft Excel 16.x was used as semi-automated support to identify predefined terms and organize records. The workbook metadata reports Microsoft Excel AppVersion 16.0300. Excel formulas did not make autonomous eligibility decisions, and no record was finally excluded without human review.
**9**	**Data collection process**	Data extraction and verification were performed independently in duplicate using rotating author roles: one author extracted the data from each included report and a second author independently verified the extracted information. All three authors participated in extraction or verification across the included studies. Discrepancies were resolved by consensus and, when necessary, by consultation with the third author. Study investigators were not contacted for additional or confirmatory information. Microsoft Excel was used to organize the extraction dataset and perform programmed conversions; all converted values and derived effect-size inputs were checked by the authors. The meta-analysis, meta-regression, and sensitivity analyses were conducted in R through RStudio.
**11**	**Study risk-of-bias assessment**	Risk of bias or methodological quality was assessed using design-appropriate tools: AXIS for cross-sectional studies, ROBINS-E for the longitudinal non-randomized exposure study, and AACODS for structured conference abstracts or grey-literature reports; STROBE/STREGA criteria were used as reporting-oriented support where applicable. Two reviewers independently assessed each included study. Disagreements were resolved by consensus and, if required, by adjudication by the third author. No automated tool assigned risk-of-bias judgments; all final judgments were made exclusively by the reviewers.
**15**	**Certainty assessment methods**	No formal certainty-of-evidence framework, such as GRADE or another structured system, was applied. Confidence in the findings was appraised narratively, considering the small number of independent studies, methodological heterogeneity, risk of bias, imprecision, dependence among multiple effect sizes, and the exploratory nature of the quantitative synthesis.

PRISMA item	Topic	Supplementary reporting information
**16b**	**Reports excluded after apparent eligibility**	Following screening, 368 potentially relevant reports were sought for retrieval. Eight reports could not be retrieved, and 360 reports were assessed for eligibility. After full-text eligibility assessment, 353 reports were excluded: 349 did not examine a relevant problematic or stressful digital exposure in relation to epigenetic or DNA methylation outcomes; two were non-human studies; one was a letter to the editor without original extractable data; and one was available only as an abstract with insufficient information to confirm eligibility or extract the required data. Supplementary Table S6 cites reports that could reasonably appear eligible and records the primary reason for exclusion. Seven studies met the expanded eligibility criteria and were included in the systematic review; four provided quantitative data suitable for the exploratory multilevel meta-analysis.
**22**	**Certainty of evidence**	Because no formal certainty framework was applied, no formal outcome-level certainty ratings are presented. The evidence is characterized narratively as preliminary, considering the small number of independent studies, substantial methodological and statistical heterogeneity, moderate overall risk of bias, imprecision, and the exploratory nature of the synthesis.
**24b**	**Protocol access**	The protocol was registered in PROSPERO under registration CRD420261431295 and is available at: https://www.crd.york.ac.uk/PROSPERO/view/CRD420261431295.
**24c**	**Protocol amendments**	No amendments to the registered eligibility criteria, outcomes, or planned synthesis were reported by the review team during preparation of this addendum. The completed checklist and this addendum therefore report no protocol amendments.
**25**	**Support and role of the funder**	Universidad Santiago de Cali funded the publication costs of the study. The support was limited to publication funding; the institution had no role in study design, searching, study selection, data extraction, risk-of-bias assessment, statistical analysis, interpretation of results, manuscript preparation, or the decision to submit the work for publication.
**26**	**Competing interests**	The authors declare that they have no commercial, financial, or other competing interests that could be construed as influencing the review.
**27**	**Availability of data, code, and other materials**	The supplementary extraction table contains all data used in the analyses. Supplementary materials provide the complete harmonized effect-size dataset, influence diagnostics, critical and risk-of-bias assessments, bootstrap analyses by tissue type, and publication-bias analyses. The R code used for the meta-analysis is preserved and is available from the corresponding review team upon reasonable request by institutional email to the lead author (hector.cabezas02@usc.edu.co). No participant-level personal or sensitive data are included in the review dataset.

Reporting note regarding PRISMA item 16b. Aggregate exclusion categories are reported in the PRISMA flow diagram. Supplementary Table S6 below additionally cites reports that could reasonably appear eligible and documents the primary reason for their exclusion, thereby providing the citation-level clarification requested by PRISMA 2020 item 16b.

SUPPLEMENTARY TABLE S6

Reports of apparent eligibility excluded after assessment and primary reason

The PRISMA flow diagram presents the aggregate reasons for all 353 exclusions after eligibility assessment. This table cites the reports identified during the audit that could reasonably be mistaken for eligible studies because their titles or abstracts refer to digital exposure, epigenetic or DNA methylation outcomes, youth, or social networks.

No.	Report	Year	DOI / identifier	Primary reason for exclusion	Eligibility criterion not met
1	Batool ST, Ali U, Mahato RK. Adolescent social-media exposure and cortical thinning signals: latest 2025 MRI-epigenome correlations.	2026	10.1097/MS9.0000000000004645	Letter to the editor; it does not report an original primary study with extractable participant-level or effect-size data.	Ineligible publication type and absence of original empirical data.
2	Malik S, Chourasia A, Pradhan RK. Prolonged Effects of Radiofrequency Electromagnetic Radiation Emanated from Mobile Phone on Global DNA Methylation and Spawning in Zebrafish, Danio rerio.	2021	Scopus record; no DOI identified in the audited workbook	Experimental study conducted in zebrafish rather than human participants.	Non-human population.
3	Immunoepigenetic-gut microbiome axis in the social networks of health disparate youth.	2019	Project/funding record; no article DOI identified	Project record rather than a report of original study results; “social networks” refers to community/interpersonal networks rather than digital social-media exposure.	Ineligible report type and no relevant digital exposure.

Note. The remaining exclusions are summarized in the flow diagram: 349 reports without a relevant problematic or stressful digital exposure linked to epigenetic or DNA methylation outcomes, two non-human reports, one letter to the editor, and one abstract with insufficient data. Table S6 focuses on reports that could reasonably appear eligible, as required by PRISMA 2020 item 16b.
